# Hypomethylation-mediated upregulation of PHOX1 promotes gastric cancer progression via transactivation of NGFR

**DOI:** 10.1038/s41420-025-02811-3

**Published:** 2025-11-28

**Authors:** Yanyan Li, Weiwei Liu, Lisheng Zheng, Guifang Zhu, Xuexia Qian, Rui Zeng, Yangwei Xu, Weiye Huang, Yongjun Huang, Qingling Zhang

**Affiliations:** 1 Department of Pathology, School of Medicine, South China University of Technology, Guangzhou, China; 2 Department of Pathology, Guangdong Provincial People’s Hospital (Guangdong Academy of Medical Sciences), Southern Medical University, Guangzhou, Guangdong China; 3 Guangdong Provincial Key Laboratory of Artificial Intelligence in Medical Image Analysis and Application, Guangzhou, China; 4 Department of Pathology, Zhujiang Hospital, Southern Medical University, Guangzhou, China; 5 State Key Laboratory of Cancer Biology, Department of Pathology, School of Basic Medicine, Xijing Hospitaland, Fourth Military Medical University, Xi’an, Shaanxi Province China

**Keywords:** Gastric cancer, Cell invasion

## Abstract

Gastric cancer (GC) remains a leading cause of global cancer-related mortality with limited therapeutic options, and its molecular mechanisms are incompletely understood. Through integrative analysis of TCGA and GEO datasets, coupled with clinical cohort validation, we identified frequent overexpression of the transcription factor PHOX1 in GC tissues, which correlated significantly with advanced T/M stages and poor patient survival. We demonstrated that *PHOX1* promoter hypomethylation, particularly at the CpG site cg04123776, drives its overexpression in GC. Functional assays revealed that overexpression of PHOX1 enhanced GC cell proliferation, migration, and invasion in vitro, while knockdown of PHOX1 inhibited these malignant behaviors. Additionally, orthotopic xenograft models confirmed its pro-metastatic role in promoting liver metastasis of GC cells. Mechanistically, RNA sequencing, chromatin immunoprecipitation assays, and luciferase reporter assays demonstrated that PHOX1 directly activated Nerve Growth Factor Receptor (NGFR) transcription. Rescue experiments with siRNA against NGFR and an ERK1/2 inhibitor further established that PHOX1 drove malignant phenotypes via NGFR and downstream ERK1/2 signaling. In conclusion, our study defines PHOX1 as a methylation-sensitive oncogene in GC, orchestrating tumor progression through transcriptional activation of NGFR, and the PHOX1-NGFR-ERK1/2 axis may serve as a therapeutic target for metastatic GC.

## Introduction

Gastric cancer (GC) continues to be one of the most prevalent and lethal malignancies worldwide, characterized by persistently limited therapeutic options and dismal survival outcomes [1]. Recent statistics from the Global Cancer Observatory highlight the disease’s substantial burden, documenting over 1 million new cases and approximately 700,000 deaths annually [2]. Despite advances in diagnostic and therapeutic modalities, the 5-year survival rate for advanced-stage GC remains poor, with metastasis accounting for nearly 90% of cancer-related mortality [3, 4]. This clinical challenge underscores the urgent need to identify novel molecular drivers of GC progression and metastatic dissemination.

The paired-related homeobox 1 (PHOX1), also known as PRRX1, is a key regulator of embryonic development and cell differentiation [5, 6]. It has recently emerged as a context-dependent modulator of tumor biology. Accumulating evidence indicates that PHOX1 influences cancer progression through diverse mechanisms, including epithelial-mesenchymal transition [7], maintenance of tumor stemness [8], and regulation of angiogenesis [9]. Notably, its functional role is cancer-type specific: PHOX1 acts as a metastasis-promoting oncogene in osteosarcoma by activating TGF-β and Wnt/β-catenin pathways [10] and sustains glioma-initiating cells via DRD2 transactivation [11], whereas it exhibits tumor-suppressive activity in clear cell renal cell carcinoma by inhibiting vascular mimicry [12]. In GC, although multiple studies have linked PHOX1 overexpression to advanced tumor stages and poor prognosis [13–15], its precise regulatory mechanisms and downstream effectors remain poorly characterized, particularly with respect to epigenetic control and GC-specific signaling networks.

Epigenetic modifications play pivotal roles in tumorigenesis and cancer progression [16]. Among these, DNA methylation is a well-characterized epigenetic mechanism that regulates gene expression by altering chromatin conformation, DNA structural stability, and protein-DNA interactions without modifying nucleotide sequences [17, 18]. Hypermethylation of CpG islands (CGIs) in gene promoters commonly induces transcriptional silencing of tumor suppressor genes, whereas hypomethylation in the same regions can drive oncogene activation [17]. The reversible and dynamic nature of DNA methylation has positioned it as a promising therapeutic target in cancer, with extensive research currently aimed at reversing promoter hypermethylation of tumor suppressor genes [18]. Although PHOX1 has been shown to exert oncogenic functions in multiple malignancies, its epigenetic regulation in tumor contexts remains poorly characterized. Whether PHOX1 expression is modulated by promoter methylation and how its epigenetic status associates with cancer prognosis remain unclear, warranting further investigation.

Here, we demonstrate that PHOX1 is epigenetically activated in GC through promoter hypomethylation at the CpG site cg04123776, with its expression levels significantly associated with advanced T/M stages and poor patient survival. Mechanistically, PHOX1 drives GC cell proliferation, invasion, and metastasis by transcriptionally activating Nerve Growth Factor Receptor (NGFR), thereby engaging the oncogenic ERK1/2 signaling cascade. These results not only establish PHOX1 as a methylation-sensitive oncogene in GC but also identify the PHOX1-NGFR-ERK1/2 axis as a promising therapeutic target. Our findings provide critical insights for developing diagnostic/prognostic biomarkers and precision therapeutic strategies in GC management.

## Results

### PHOX1 is upregulated in GC and correlated with poor patient survival

**Fig. 1:**
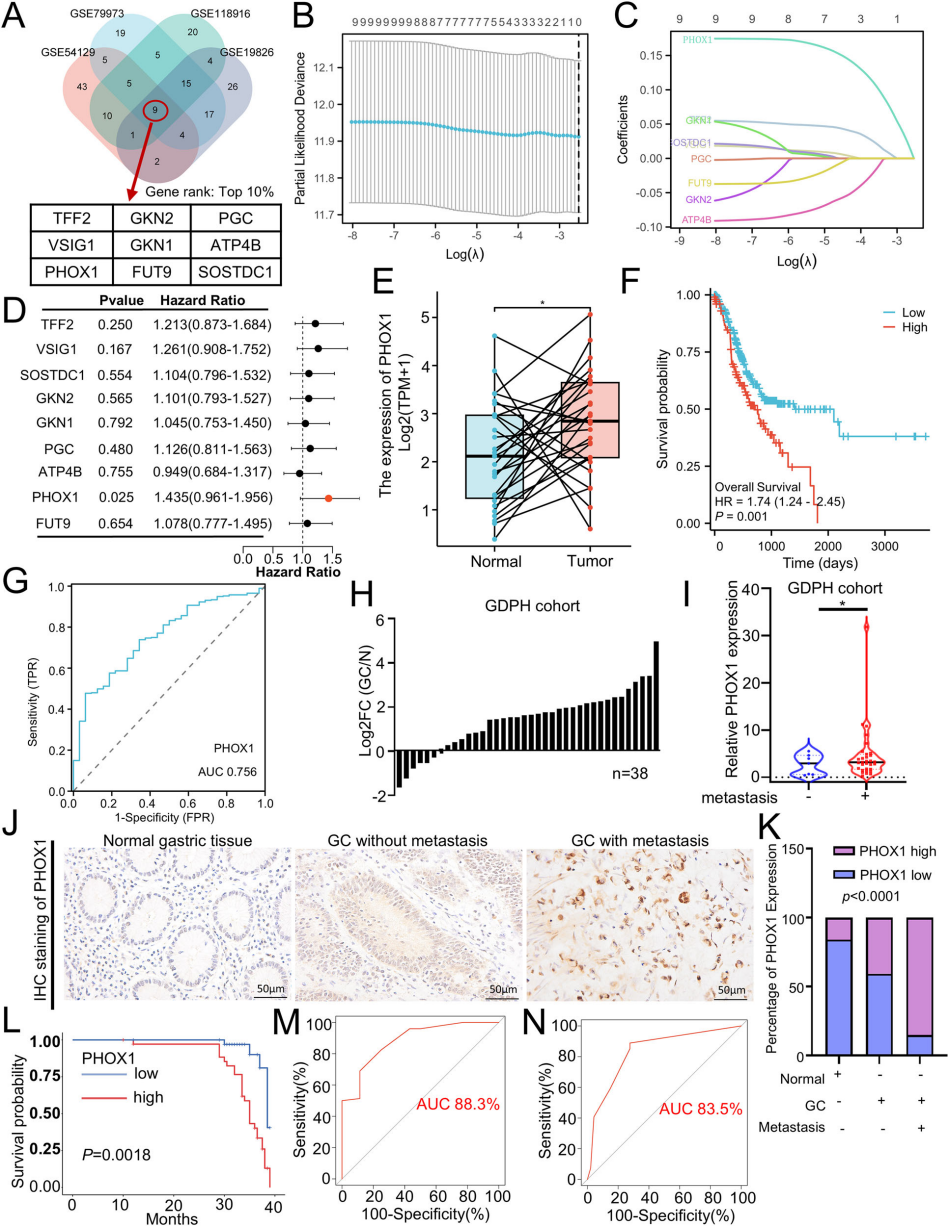
Upregulated PHOX1 is correlated with poor survival in GC. **A** Venn diagram illustrating the overlap of differentially expressed genes (DEGs) between GC and normal gastric tissues across four GEO datasets (GSE54129, GSE79973, GSE118916, and GSE19826). **B** Plot illustrating the partial likelihood deviance in LASSO regression, with the *x*-axis representing log(λ) and the *y*-axis showing partial likelihood deviance using the TCGA_STAD dataset. **C** Curve plot showing the coefficients of variables (PHOX1, GKN1, SOSTDC1, PGC, FUT9, GKN2, ATP4B, TFF2, VSIG1) against log(λ) in LASSO regression using the TCGA_STAD datase. **D** DEGs-based signature for prognostic prediction of GC patients in the TCGA_STAD dataset. **E** Bar chart illustrating PHOX1 expression in GC and matched adjacent normal gastric tissues from the TCGA_STAD dataset. **F** Survival analysis of PHOX1 expression using the TCGA_STAD dataset. **G** Receiver operating characteristic (ROC) curves for PHOX1 expression in the TCGA_STAD dataset. **H** Ratio (GC/N) of PHOX1 mRNA expression in 38 pairs of GC and adjacent normal mucosal tissues, determined by RT-qPCR. Expression levels were normalized to GAPDH. **I** PHOX1 mRNA expression in GC tissues with or without metastasis, detected by RT-qPCR (*n*[with metastasis] = 31, *n*[without metastasis] = 7). Metastasis includes distant and lymph node metastasis. **J** PHOX1 protein expression in GC tissues was detected by IHC. scale bars: 50 μm. **K** Percentage of GC samples with high/low PHOX1 expression, categorized by metastatic status in GC tissues and normal gastric mucosa. **L** Kaplan–Meier curves for overall survival based on PHOX1 expression. Cutoff value was determined by stratified optimal cut-off analysis (*n*[low] = 39, *n*[high] = 35). **M** Area under the ROC curve for PHOX1 in the differential diagnosis of gastric mucosa and GC. **N** Area under the ROC curve for PHOX1 in GC tissues with metastasis or without metastasis. *P*-value is determined by *t*-test. *P < 0.05.

To systematically identify genes associated with GC progression, we performed differential gene expression (DEGs) analysis across four independent datasets (GSE54129, GSE79973, GSE118916, and GSE19826) using the limma package in R. Applying stringent criteria (|log2FC| > 2, adjusted *p*-value < 0.05), we identified nine consistently dysregulated genes (TFF2, VSIG1, SOSTDC1, GKN2, GKN1, PHOX1, PGC, ATP4B, FUT9) across all four datasets (Figs. 1A and S1A). Subsequently, LASSO regression and univariate Cox analysis collectively identified PHOX1 as the most significant predictor of GC progression (Fig. 1B–D).

Pan-cancer analysis using SangerBox (http://sangerbox.com/) revealed context-dependent PHOX1 expression patterns, with significant upregulation in 12 tumor types (including GC) but downregulation in 5 other tumor types (Fig. S1B), suggesting its dual oncogenic/anti-oncogenic roles. In the TCGA_STAD dataset, PHOX1 expression was markedly elevated in tumor tissues compared with paired normal tissues (Fig. 1E), and patients with high PHOX1 expression exhibited a markedly shorter overall survival (Fig. 1F). Receiver Operating Characteristic (ROC) analysis showed that the area under the curve of PHOX1 was 0.756, indicating that PHOX1 has the potential to serve as a diagnostic indicator for GC (Fig. 1G). Besides, clinicopathological analysis revealed progressive PHOX1 upregulation with advancing T stages (Fig. S1C), advanced metastatic status (Fig. S1D), and pathological stages (Fig. S1E), though no association was observed with N stages, gender, or age groups (Fig. S1F–H).

RT-qPCR analysis of freshly collected GC tissues and adjacent normal mucosal tissues from our institutional cohort confirmed significant PHOX1 overexpression in GC tissues (Fig. 1H), with further upregulation in metastatic GC tissues (Fig. 1I). IHC staining revealed stronger nuclear PHOX1 expression in GC tissues, particularly in metastatic lesions (Fig. 1J–K). Additionally, patients with higher PHOX1 expression (defined using the optimal cut-off value) showed significantly shorter overall survival (Fig. 1L). Clinicopathological characteristics are detailed in Supplementary Table S1, which revealed that tumor depth, lymph node metastasis, distant metastasis, histological grade, and tumor stage differed significantly according to PHOX1 expression levels. Moreover, ROC curve analysis revealed robust diagnostic performance for PHOX1 in distinguishing GC tissues from adjacent normal mucosal tissues and metastatic from non-metastatic GC cases (Fig. 1M, N). These findings demonstrate that PHOX1 is upregulated in metastatic GC, suggesting its potential as a prognostic indicator for GC patients.

### Promoter hypomethylation drives oncogenic upregulation of PHOX1 in GC

**Fig. 2:**
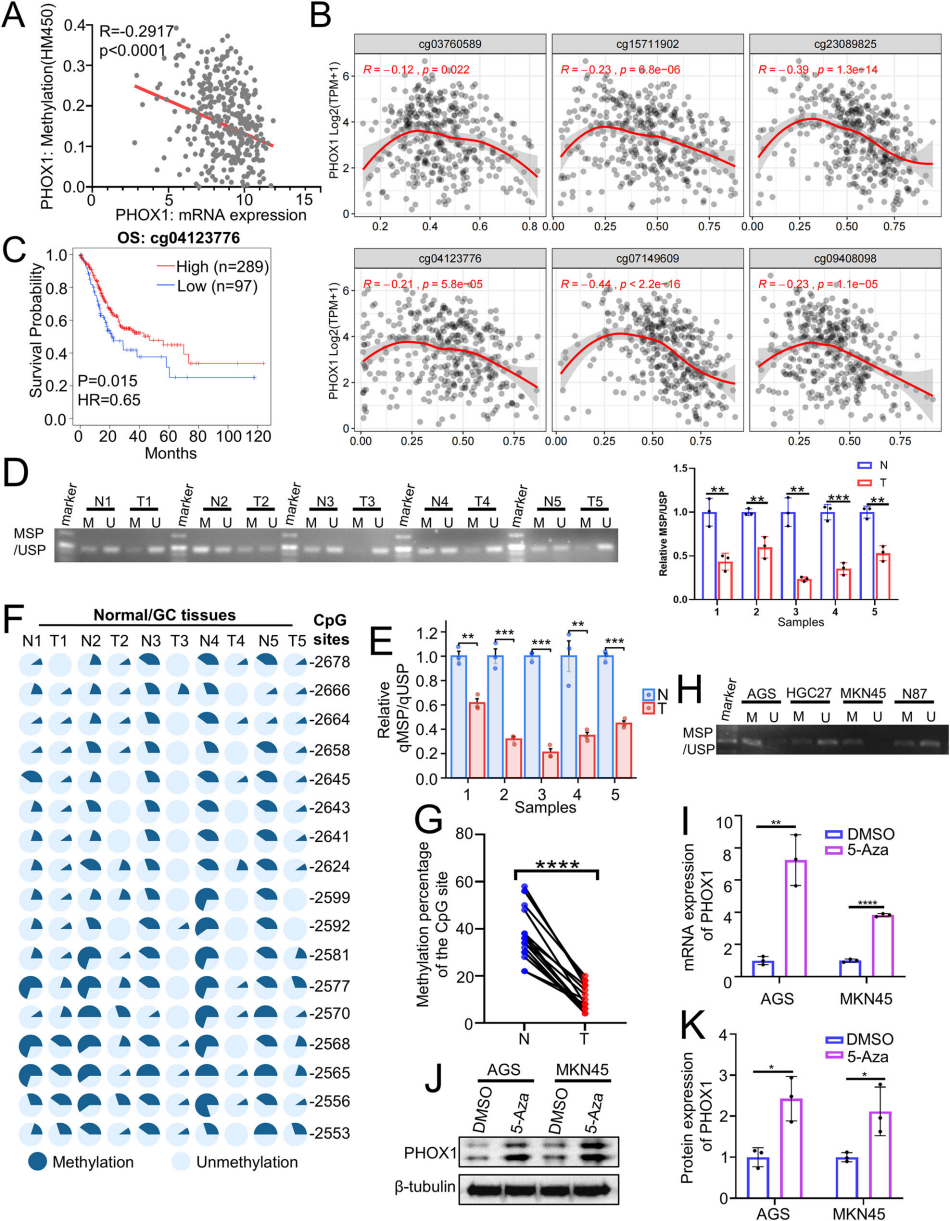
Promoter hypomethylation contributes to PHOX1 upregulation in GC. **A** Correlation analysis of PHOX1 mRNA expression and *PHOX1* promoter methylation using the TCGA_STAD dataset. **B** Correlation analysis between PHOX1 and PHOX1-related CpG sites using the SMART App. **C** Overall survival curves based on methylation status of the cg04123776 CpG site using the TCGA_STAD dataset. **D** Representative gel images of MSP results for 5 pairs of GC tissues and adjacent normal mucosal tissues. Primers were designed to be specific for unmethylated (U) or methylated (M) DNA (left panel); bar graphs (right panel) show the band intensity analysis of gel images, expressed as the relative ratio of methylated (M) to unmethylated (U) band intensity, which was normalized to paired adjacent normal mucosal tissues. **E** Bar chart showing CpG methylation status (relative qMSP/qUSP) in the *PHOX1* promoter—determined by qMSP and qUSP—in paired GC tissues (T) and adjacent normal mucosal tissues (N). **F**, **G** Pie charts (**F**) and line graphs (**G**) showing methylation status of the targeted *PHOX1* promoter region (including cg04123776; −2716 to −2543 bp relative to the TSS) for GC tissues and adjacent normal mucosal tissues. Gray-blue and navy-blue indicate unmethylated and methylated CpGs, respectively. **H** MSP/USP analyses showing methylation status of the *PHOX1* promoter in GC cells. **I–K** Levels of PHOX1 mRNA and protein in AGS and MKN45 cells treated with or without 5-aza-2’-deoxycytidine. *P*-value is determined by *t*-test. *P < 0.05, **P < 0.01, ***P < 0.001, ****P < 0.0001.

To elucidate the epigenetic regulation of PHOX1, we first analyzed the TCGA_STAD dataset and identified a significant inverse correlation between *PHOX1* promoter methylation and PHOX1 mRNA level (Spearman’s correlation coefficient, *r* = −0.2917, *P* < 0.0001; Fig. 2A). We then analyzed the *PHOX1* promoter sequence (−3000 to +500 bp relative to the transcription start site, TSS) using MethPrimer (https://methprimer.com/), a tool for CpG island prediction and methylation PCR primer design, identifying two CGIs and multiple CpG sites within this region (Fig. S2A). Computational analysis using SMART_App (https://bio.tools/SMART_App) revealed six CpG sites in the *PHOX1* promoter region (cg03760589, cg15711902, cg23089825, cg04123776, cg07149609, and cg09408098) that showed strong negative correlations with PHOX1 mRNA expression (Figs. 2B and S2B). Validation in the GSE164988 dataset (12 pairs of matched tumor-normal tissues from GC patients) confirmed significant hypomethylation at the cg23089825 and cg04123776 sites in tumor tissues compared with their matched normal tissues (Fig. S2C). Besides, survival analysis of these six CpG sites revealed that hypomethylation at cg04123776 and cg09408098 predicted poorer overall survival (Figs. 2C and S2D). Notably, cg04123776 emerged as the more clinically relevant site, exhibiting a stronger negative correlation with PHOX1 expression, the highest hypomethylation level in tumor tissues, and more significant prognostic value. Therefore, we hypothesized that hypomethylation at the cg04123776 CpG site may serve as a key regulatory mechanism underlying PHOX1 upregulation in GC.

To validate the role of hypomethylation at the cg04123776 CpG site in PHOX1 expression, we performed targeted methylation analysis of the cg04123776 locus (−2706 to −2533 bp relative to TSS) in randomly selected matched tumor/normal tissue pairs. The Methylation-specific PCR (MSP), Unmethylation-specific PCR (USP), and Bisulfite Sequencing PCR (BSP) products, along with the location of cg04123776 in the *PHOX1* promoter region, are presented in Supplementary Fig. S2A. Qualitative assays, including MSP and USP, were first used to confirm the presence of differential PHOX1 methylation between tumor tissues and adjacent normal mucosal tissues, with tumor tissues showing weaker signals of methylated *PHOX1* promoter alleles (Fig. 2D). Subsequently, methylation-specific quantitative real-time PCR (qMSP) was performed to validate these observations, which consistently showed a significantly lower percentage of methylated PHOX1 alleles in tumor tissues compared to adjacent normal mucosal tissues (Fig. 2E). Additionally, BSP revealed striking PHOX1 hypomethylation in tumor tissues versus normal tissues (Fig. 2F–G). Consistent with the clinical observations, PHOX1-high cells (HGC27 and N87) showed marked hypomethylation of PHOX1 at this locus, while PHOX1-low cells (AGS and MKN45) maintained hypermethylation (Fig. 2H). Furthermore, to determine direct epigenetic regulation of PHOX1 expression by promoter methylation, we treated AGS and MKN45 cells with the DNA methyltransferase inhibitor 5-aza-2′-deoxycytidine (5-Aza), and found elevated PHOX1 mRNA and protein levels upon 5-Aza treatment (Fig. 2I–K). These results provide mechanistic evidence that DNA hypomethylation at cg04123776 drives PHOX1 overexpression in GC.

### PHOX1 promotes proliferation and motility of GC cells in vitro

**Fig. 3:**
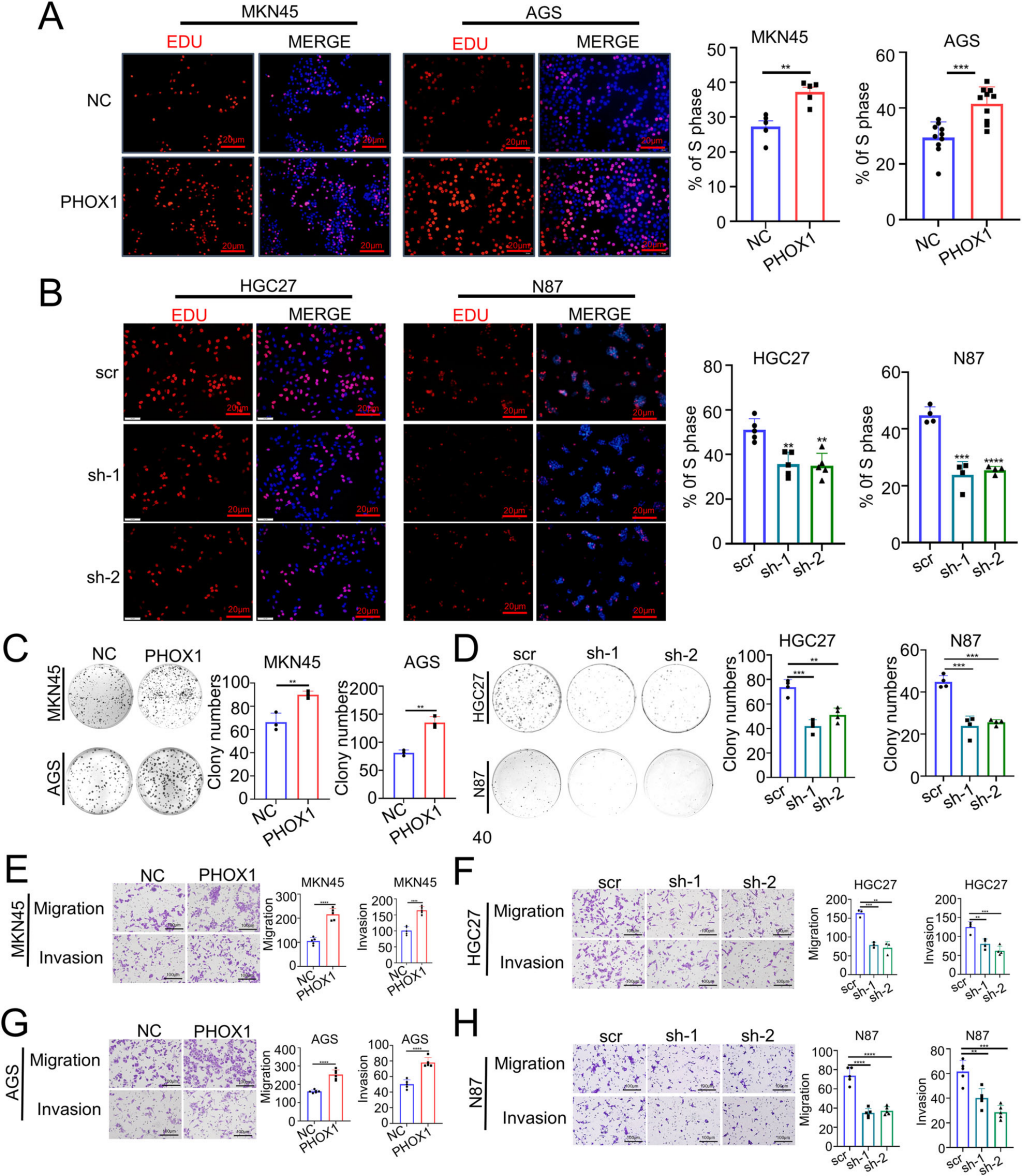
PHOX1 promotes proliferation and motility of GC cells in vitro. **A**, **B** Proliferative ability of GC cells following PHOX1 overexpression or knockdown, detected by 5-ethynyl-2′-deoxyuridine (EdU) assay (**A**, **B**). Scale bar: 20 μm. **C**, **D** Representative images of clone formation and statistics of colony counts in GC cells with PHOX1 overexpression or knockdown. **E–H** Microscopic images and quantification of the migration and invasion of GC cells. *P*-value is determined by *t*-test. **P < 0.01, ***P < 0.001, ****P < 0.0001.

To investigate the oncogenic role of PHOX1 in GC progression, we first characterized its expression patterns in GC cell lines. Western blot and RT-qPCR analyses revealed differential PHOX1 expression, with relatively low levels in AGS and MKN45 cells compared with those in HGC27 and N87 cells (Fig. S3A, B). We subsequently established isogenic cell models through stable transfection: PHOX1-overexpressing AGS_PHOX1 and MKN45_PHOX1 cells, and PHOX1-knockdown HGC27_shPHOX1 and N87_shPHOX1 cells (Fig. S3C–F). In proliferation assays, PHOX1 overexpression increased cell growth and colony-forming capacity of AGS and MKN45 cells, while PHOX1 knockdown attenuated these capacities in HGC27 and N87 cells (Fig. 3A–D). Besides, Transwell migration and invasion assays revealed that PHOX1 overexpression enhanced cell migration and invasion, whereas PHOX1 knockdown impaired these cell motilities (Fig. 3E–H). These findings suggest that PHOX1 markedly promotes the proliferative, migratory, and invasive capabilities of GC cells.

### PHOX1 promoted growth and metastasis of GC cells in vivo

**Fig. 4:**
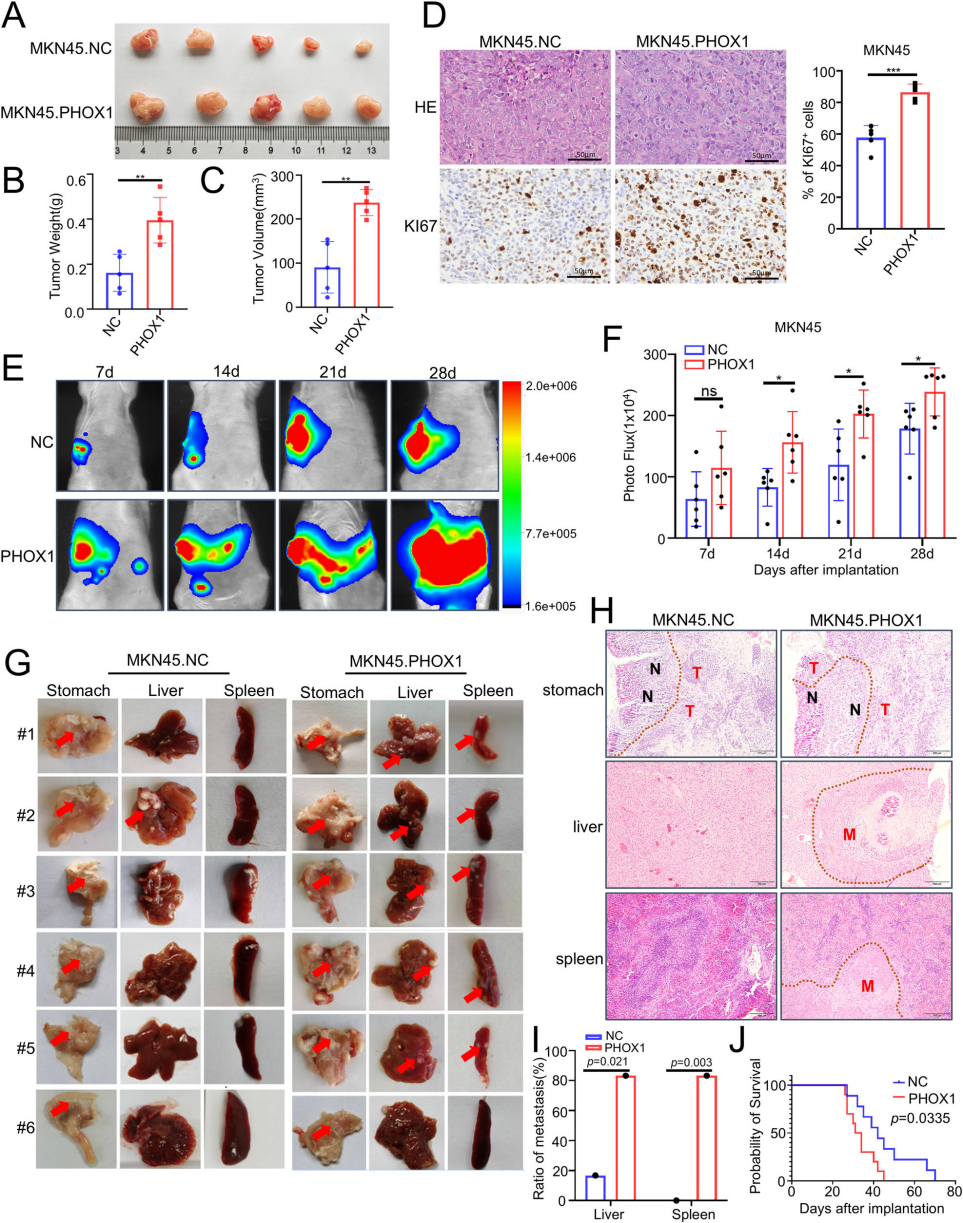
PHOX1 promotes growth and metastasis of GC cells in vivo. **A** Gross images of subcutaneous tumors formed by PHOX1-overexpressing GC cells. *n* = 5 per group. **B**, **C** Weight and volume analyses of subcutaneous tumors. *n* = 5. **D** Representative H&E staining and IHC staining of Ki67 in subcutaneous tumors formed by the indicated cells. Scale bars: 50 μm. **E** Representative bioluminescent images of xenografts after tumor cell implantation MKN45-Luc cells stably transfected with NC or PHOX1 vectors were implanted into mouse stomachs. **F** The fluorescence signal intensity of xenografts in all groups. **G** Images of gross mice GC orthotopic tumors, livers, and spleens formed by the indicated cell lines. **H** Representative images of GC orthotopic tumors, livers, and spleens with H&E staining. Scale bars: 100 μm. **I** Histogram showing statistics of liver and spleen metastasis rates in mice, *p*-value conducted by chi-square test. **J** Kaplan–Meier survival analysis of mice bearing xenografts. *n* = 6 for each group. *P < 0.05, **P < 0.01, ***P < 0.001, ns no significance.

To evaluate the oncogenic potential of PHOX1 in GC cells in vivo, multiple xenograft models were established using GC cells with modulated PHOX1 expression. In subcutaneous xenografts, xenografts with PHOX1 overexpression exhibited significantly accelerated growth kinetics compared to those in the control group (Fig. 4A–C). Histopathological analysis using hematoxylin and eosin (H&E) and Ki-67 IHC staining confirmed significantly enhanced proliferative activity in PHOX1-overexpressing tumors (Fig. 4D).

Using an orthotopic implantation model with luciferase-labeled MKN45 cells, bioluminescence intensity in the PHOX1 overexpression group was significantly stronger than that in the control group, with significantly increased metastatic foci formation in the PHOX1 overexpression group (Fig. 4E, F). Besides, gross examination of the stomach, liver, and spleen, combined with H&E staining, demonstrated that tumors in the PHOX1 overexpression group were significantly larger and the tumor cells exhibited a more invasive morphology, with significantly more metastatic nodules in the livers and spleens (Fig. 4G–I). Additionally, Kaplan*–*Meier (KM) survival analysis of tumor-bearing nude mice showed that the group injected with MKN45_PHOX1 cells exhibited significantly shorter overall survival compared with the control group (Fig. 4J). These findings suggest that PHOX1 overexpression enhances the tumorigenicity and metastatic capacity of GC cells in vivo.

### PHOX1 transcriptionally upregulates NGFR

**Fig. 5:**
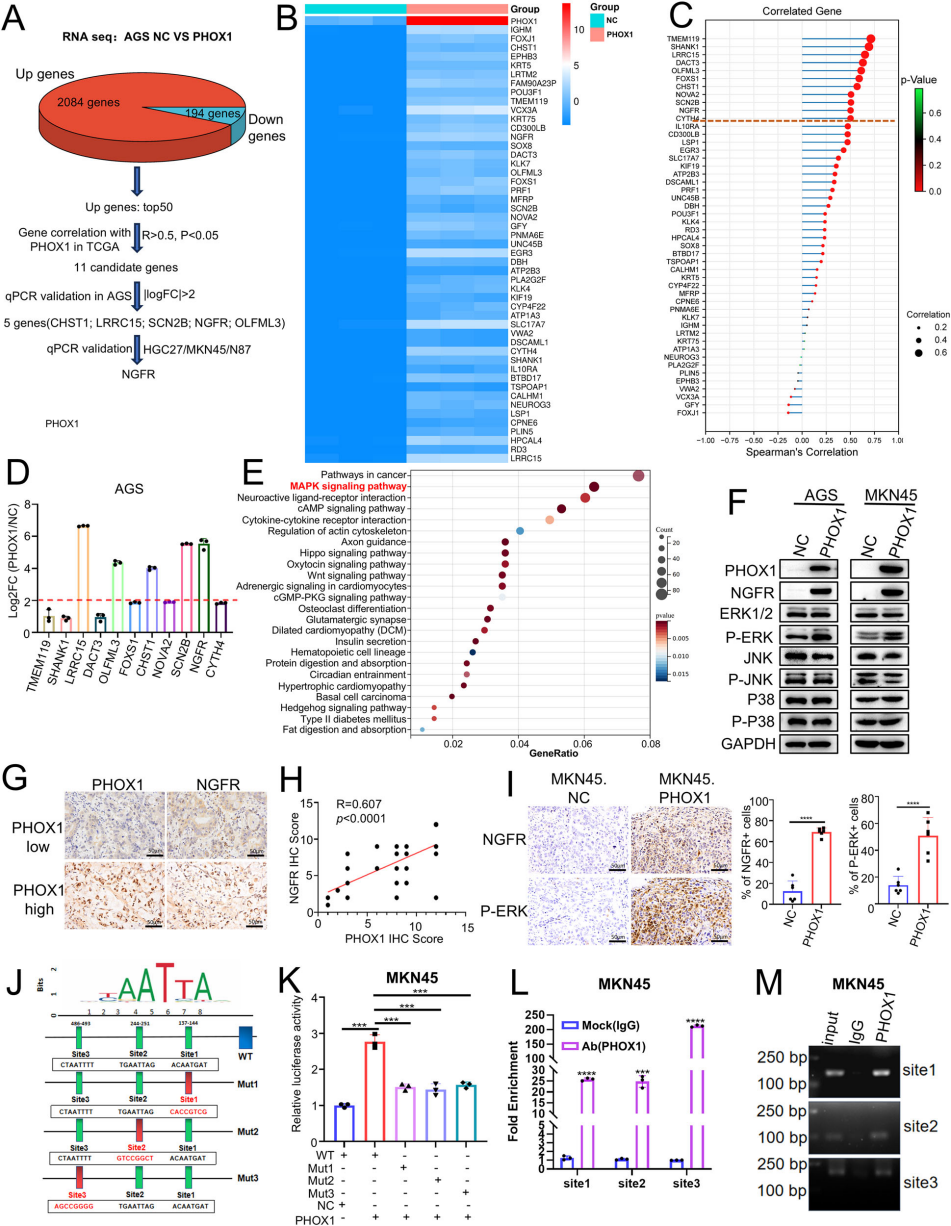
PHOX1 upregulates NGFR and activates downstream ERK1/2 signaling. **A** Schematic view of the workflow for identifying candidate downstream genes of PHOX1 in AGS cells. **B** Heatmap of the top 50 upregulated genes in AGS cells upon PHOX1 overexpression. **C** Correlations between PHOX1 and the top 50 upregulated genes in the TCGA_STAD dataset. **D** Effect of PHOX1 overexpression on expression of the candidate DEGs was detected by RT-qPCR in AGS cells. **E** Bubble plot showing KEGG pathway enrichment analysis of DEGs in AGS cells with PHOX1 overexpression compared to the control group (NC). **F** Effect of PHOX1 overexpression on the NGFR and MAPK signaling pathway detected by western blot. **G**, **H** Representative IHC staining of PHOX1 and NGFR expression in GC tissues (**G**); correlation analysis of PHOX1 and NGFR expression levels in 62 GC samples, assessed by Pearson correlation analysis (**H**). **I** Representative IHC staining of NGFR and phosphorylated ERK (P-ERK) expression in mouse GC orthotopic tumors. **J** Potential PHOX1 binding sites in the *NGFR* promoter, predicted using the JASPAR database. **K** Effect of PHOX1 on the transcriptional activity of the *NGFR* promoter. Luciferase reporter plasmids containing wild-type (WT) or mutant (Mut) *NGFR* promoter sequences were co-transfected with either the PHOX1-overexpressing plasmid or empty vector (NC) into GC cells, followed by luciferase reporter assays. **L** ChIP-qPCR analysis of PHOX1 enrichment on NGFR gene promoter. **M** Agarose gel electrophoresis of ChIP-PCR products, showing PHOX1 enrichment at PHOX1-binding sites 1, 2, and 3 on the *NGFR* promoter. Molecular weight markers are indicated alongside the gels. *P < 0.05, **P < 0.01, ***P < 0.001, ****P < 0.0001, ns no significance.

To elucidate the molecular mechanisms underlying PHOX1-mediated GC progression, RNA sequencing (RNA-seq) was performed in AGS cells overexpressing PHOX1. DEGs analysis (fold change ≥ 2) identified 2084 upregulated genes and 194 downregulated genes (Fig. S4A). Through systematic screening (Fig. 5A), the top 50 upregulated genes were selected (Fig. 5B), and their expression was correlated with PHOX1 in the TCGA_STAD dataset. Spearman correlation analysis (*r* > 0.5, *p* < 0.05) revealed 11 candidate targets (TMEM119, SHANK1, LRRC15, DACT3, OLFML3, FOXS1, CHST1, NOVA2, SCN2B, NGFR, and CYTH4; Fig. 5C), among which NGFR showed the most consistent upregulation (log2FC > 2) across multiple GC cell lines (AGS, MKN45, HGC27, N87) upon PHOX1 overexpression or knockdown (Figs. 5D and S4B–D). Therefore, NGFR was selected as the candidate downstream target gene of PHOX1.

Kyoto Encyclopedia of Genes and Genomes (KEGG) analyses of the RNA sequencing data identified MAPK signaling as a key pathway (Fig. 5E), which may serve as a downstream pathway of NGFR. Western blot analysis confirmed NGFR protein upregulation and elevated phosphorylation of ERK1/2 in PHOX1-overexpressing cells, demonstrating that PHOX1 upregulated NGFR and activated downstream MAPK signaling (Fig. 5F). Besides, bioinformatics analysis using the GEPIA2 database revealed a strong positive correlation between PHOX1 and NGFR expression in STAD samples (Fig. S4E). Moreover, IHC staining and RT-qPCR revealed that NGFR expression was elevated in PHOX1-high GC tissues (Figs. 5G, H and S4F), and NGFR expression was associated with poor patient survival (Fig. S4G). Furthermore, in the orthotopic xenograft model, a notable upregulation of NGFR and phosphorylated ERK (P-ERK) was observed in the PHOX1 overexpression group (Fig. 5I). These findings suggest that PHOX1 may upregulate NGFR and activate the ERK1/2 signaling pathway in GC cells.

Since PHOX1 is a transcription factor and regulates the transcriptional activity of target genes, we hypothesized that PHOX1 may upregulate NGFR expression by directly binding to its promoter. JASPAR (https://jaspar.elixir.no/) predictions identified three potential PHOX1 binding sites in the *NGFR* promoter (Fig. 5J). Luciferase reporter assays demonstrated PHOX1-dependent transcriptional activation at all three sites in both AGS and MKN45 cell lines (Figs. 5K and S4H). Additionally, ChIP-qPCR assay results confirmed that PHOX1 binds to these three sites (Figs. 5L, M and S4I, J), establishing NGFR as a direct transcriptional target of PHOX1.

### PHOX1 promoted the proliferation and motility of GC cells through the NGFR-ERK1/2 signaling pathway

**Fig. 6:**
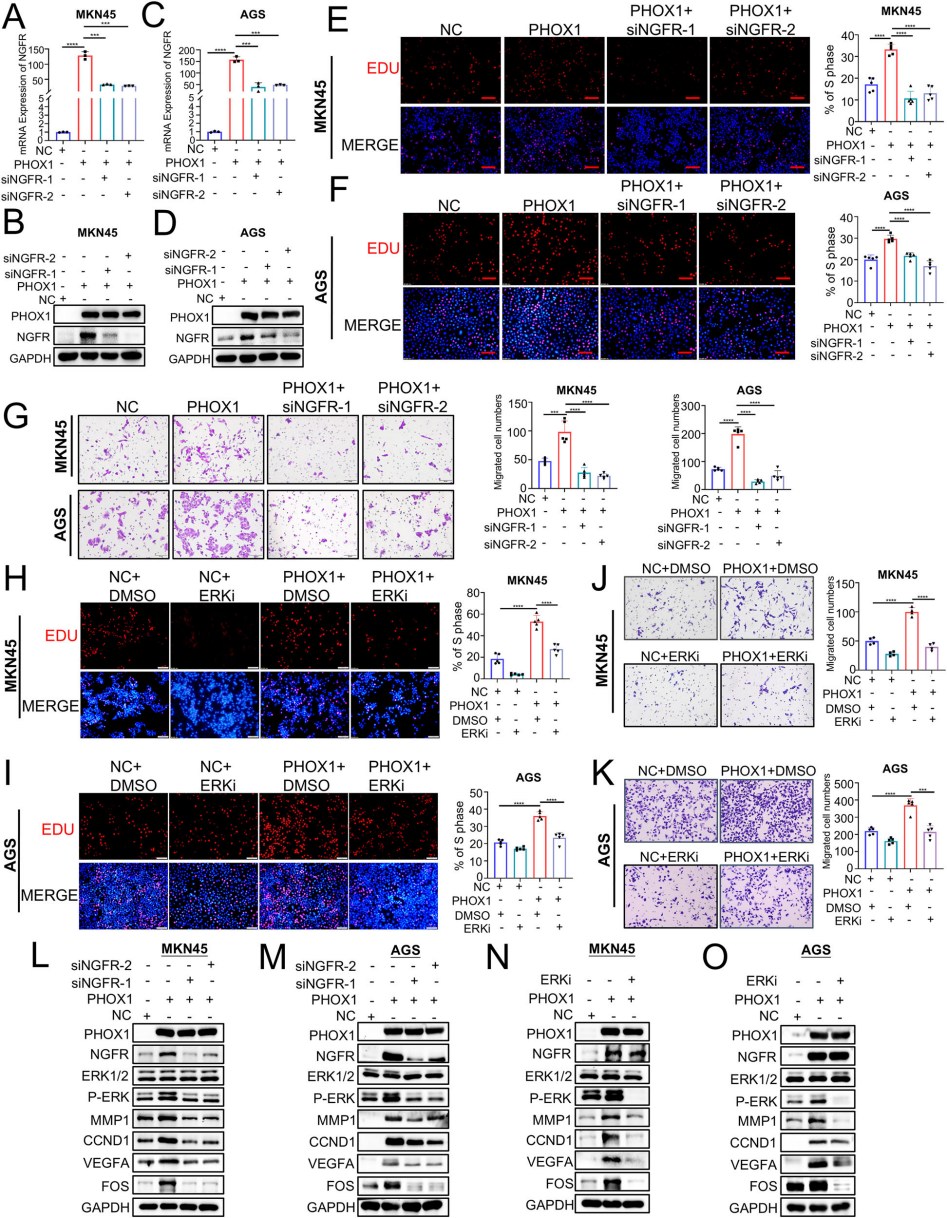
PHOX1 promotes GC cell proliferation and motility through the NGFR-ERK1/2 signaling pathway. **A–D** RT-qPCR and Western blot (WB) assays were used to verify the successful establishment of PHOX1-overexpressing and NGFR-knockdown GC cell models at 48 h after transfection. **E**, **F** Proliferative ability of PHOX1-overexpressing GC cells following NGFR knockdown, assessed by 5-ethynyl-2′-deoxyuridine (EdU) assay. **G** Transwell assay showing the effect of NGFR siRNA on the migration ability of PHOX1-overexpressing GC cells. **H**, **I** Effect of the ERK1/2 inhibitor SCH772984 (ERKi) on the proliferative ability of PHOX1-overexpressing GC cells, assessed by EdU assay. **J, K** Transwell assay showing the effect of the ERK1/2 inhibitor SCH772984 (ERKi) on the migration ability of PHOX1-overexpressing GC cells. **L–O** Western blot (WB) analysis was performed to examine the expression of key ERK1/2 pathway markers in PHOX1-overexpressing GC cells with or without NGFR knockdown or ERKi treatment. ***P < 0.001, ****P < 0.0001.

To functionally validate the role of NGFR-ERK1/2 signaling in PHOX1-mediated GC progression, we conducted comprehensive rescue experiments using siRNA against NGFR and the ERK1/2 inhibitor SCH772984 (ERKi). RT-qPCR and WB assay validated the effective knockdown of NGFR in GC cells (Fig. 6A–D). Knockdown of NGFR in GC cells significantly reversed the PHOX1-mediated promotion of cell proliferation and migration (Fig. 6E–G). Consistently, treatment with ERKi resulted in a remarkable reduction in cell proliferation and migration of PHOX1-overexpressing GC cells (Fig. 6H–K). Besides, WB assay revealed that knockdown of NGFR or treatment with ERKi dramatically inhibited the PHOX1-mediated upregulation of p-ERK, MMP1, CCND1, VEGFA, and FOS (Fig. 6L–O), crucial downstream effectors of the ERK pathway that promote tumorigenesis and metastasis in GC [19, 20]. These findings suggest that the NGFR-ERK1/2 pathway plays a crucial role in PHOX1-mediated GC progression.

## Discussion

Our study provides compelling evidence that PHOX1 functions as a critical oncogene in GC progression through an epigenetic-transcriptional regulatory axis. By integrating multiple datasets analyses and functional validation, we have delineated a novel PHOX1-NGFR-ERK1/2 signaling cascade that drives GC aggressiveness and correlates with adverse clinical outcomes. These findings significantly advance our understanding of GC pathogenesis and uncover new therapeutic opportunities.

The oncogenic role of PHOX1 has been increasingly recognized across various malignancies [13, 21–26], and our study substantially extends these observations in GC. Through a comprehensive analysis of multiple independent cohorts (GEO, TCGA, and our institutional samples), we consistently observed PHOX1 overexpression in GC tissues, which strongly correlated with advanced tumor stages and reduced patient survival. While previous studies have identified TWIST1 as a regulator of PHOX1 in renal fibrosis [27], research on the regulation of PHOX1 in tumors remains limited, and our work uncovers a distinct epigenetic mechanism underlying PHOX1 activation in GC. We have demonstrated that hypomethylation of a specific CpG site (cg04123776) in the *PHOX1* promoter region serves as a primary driver of its transcriptional upregulation. This finding is particularly significant given the established role of DNA methylation alterations in cancer development. The causal relationship between DNA hypomethylation and PHOX1 overexpression was rigorously validated through 5-aza-2′-deoxycytidine demethylation experiments. Importantly, we established the clinical relevance of this epigenetic modification by showing that PHOX1 hypomethylation correlates with poor prognosis. However, we acknowledge that the relatively small size of our clinical validation cohort represents a limitation that should be addressed in future multicenter studies; additionally, the mechanism by which hypomethylation of cg04123776 upregulates PHOX1 expression requires further clarification.

Our functional studies provide robust evidence that PHOX1 promotes GC growth and metastasis through both in vitro and in vivo models. Through systematic RNA-seq and subsequent validation, we identified NGFR as a key target and downstream effector of PHOX1. NGFR is a transmembrane receptor with well-documented roles in promoting ERK1/2 signaling activation and cancer progression [28–31]. Our findings are particularly significant as they reveal that the PHOX1-NGFR axis converges on ERK1/2 activation, a central signaling pathway in cancer biology [20]. The abrogation of PHOX1’s tumor-promoting effects by either NGFR knockdown or ERK1/2 inhibition provides compelling evidence for the functional importance of the PHOX1-NGFR-ERK1/2 signaling axis in GC progression. This finding is consistent with numerous studies demonstrating the critical role of persistent ERK1/2 activation in cancer progression [32–36], establishing PHOX1 as a novel upstream regulator of this pathway in GC progression.

The PHOX1-NGFR-ERK1/2 axis we have identified offers several promising clinical translational opportunities. First, the methylation status of the PHOX1 cg04123776 locus could serve as a valuable biomarker for patient stratification in GC and prognosis prediction. Second, the therapeutic potential of targeting this axis is supported by our findings that both genetic interference (NGFR knockdown) and pharmacological interventions (ERK1/2 inhibition) effectively abrogate PHOX1-mediated oncogenic effects in GC.

In conclusion, our study delineates a novel and complete oncogenic pathway in GC—from epigenetic alteration, through transcriptional regulation, to kinase signaling. The discovery of this PHOX1-NGFR-ERK1/2 axis not only deepens our understanding of GC biology but also paves new avenues for developing targeted therapeutic strategies against this deadly malignancy.

## Methods

### Tissue specimens and cell culture

Seventy-four GC patient samples and 44 normal gastric tissue samples were obtained from Guangdong Provincial People’s Hospital. Prior to surgery, all patients did not receive chemotherapy or radiation therapy. Written informed consent was obtained from all patients, and approval from the hospital’s Institutional Review Board was also obtained. The cell lines used in this study included GES-1, AGS, MGC803, N87, MKN45, HGC27, and HEK293T, which were obtained from the Cell Bank of the Chinese Academy of Medical Sciences (Shanghai, China). GES-1, AGS, MGC803, N87, and MKN45 cells were maintained in RPMI-1640 medium, while HGC27 and HEK293T cells were cultured in DMEM medium; both media were supplemented with 10% fetal bovine serum (FBS; Sigma-Aldrich, St. Louis, MO, USA; catalog number: F0193). All cells were incubated at 37 °C in a humidified atmosphere with 5% CO₂.

Regular mycoplasma tests were performed on all cell lines, and all results were negative for contamination. Specifically, mycoplasma testing was performed using the Myco-Lumi™ Luminescence Mycoplasma Detection Kit (high-sensitivity instrument compatible; Beyotime Biotechnology, Catalog No. C0298S), with the procedure strictly following the manufacturer’s instruction manual.

### TCGA data analysis and patient overall survival analyses

The bioinformatics analyses of The Cancer Genome Atlas (TCGA) data were performed using the online platform Xiantao (https://www.xiantaozi.com/), a comprehensive toolbox that is integrated with standardized pipelines for high-throughput omics data processing and survival analysis.

Survival analyses were conducted using the “Survival Analysis” module in Xiantao. GC patients were stratified into high- and low-expression groups based on the optimal cut-off value of PHOX1 mRNA expression levels. KM survival curves were generated to visualize the difference in overall survival (OS) between groups, and the log-rank test was applied to assess the statistical significance, with *P* < 0.05 considered statistically significant.

### Construction of overexpression or knockdown plasmids, transfection, and establishment of stable cell lines

The shRNAs against PHOX1 were designed based on the complementary DNA (cDNA) sequence of the PHOX1 gene and expressed through the lentiviral vector pLVX-puro purchased from Geneyuan Co., Ltd. (Guangzhou, China). The target sequences were as follows: shRNA-1: 5′-GGAATAGGACAACCTTCAA-3′; shRNA-2: 5′-CACGTGACACGTTCGGAGA-3′; and the negative control (shNC) used the sequence 5′-TTCTCCGAACGTGTCACGT-3′. The lentiviral vector pLVX-puro was purchased from Geneyuan Co., Ltd. (Guangzhou, China). For PHOX1 overexpression, the coding sequence (CCDS) of the PHOX1 gene was inserted into the lentiviral expression vector pLVX-3 × FLAG-Puro (purchased from Geneyuan Co., Ltd.), and the insertion was performed using Clone Enzyme Mix (ThermoFisher Scientific, USA) to construct the pLVX-PHOX1-3 × FLAG expression plasmid.

For lentivirus production, the pLVX plasmids were co-transfected into HEK293T cells with the packaging vectors psPAX2 and pMD2.G at a ratio of 4:3:1 using Lipofectamine 2000 Transfection Reagent (Invitrogen, USA). The culture medium was replaced with fresh medium 12 h after transfection. After an additional 48 h of cell culture, the culture supernatant containing lentiviruses was collected, depleted of cell debris by centrifugation at 3000 rpm at 4 °C for 10 min, and subsequently filtered through a 0.45 μm PVDF filter (Cat. No.: FPE404000; JETBIOFIL Scientific Co., Ltd., China). The lentiviral solutions were stored at −80 °C until use.

For the establishment of stable PHOX1 overexpression or knockdown GC cell lines, the GC cells were infected with the prepared lentiviral solutions in the presence of 8 μg/mL polybrene (Beyotime Biotechnology, Cat. No.: ST1380, China) for 24 h, and the medium was replaced with fresh medium for an additional 24 h of culture. Lentivirus-infected cells were then selected using 2 μg/mL puromycin for 72 h. The plasmids pLVX-3 × FLAG-luci-Puro and pLVX-shNC-puro were used as the overexpression negative control (NC) and knockdown negative control, respectively.

### Synthesis and transfection of NGFR small interfering RNA (siRNA)

For NGFR gene silencing, two specific siRNA oligonucleotides (si-1 and si-2) were designed using an online algorithm from Santa Cruz Biotechnology, which targets the human NGFR mRNA sequence (NM_002507.4). The target sequences were: siNGFR-1: 5′-GACAAGCAGAACACCGTGT-3′; siNGFR-2: 5′-CGTTGGATTACACGGTCCA-3′. All siRNAs were synthesized by Geneyuan Co., Ltd. (Guangzhou, China) using standard desalting purification. To avoid off-target effects, each sequence was validated using the BLAST program against the human genome to ensure specificity. A non-targeting siRNA (si-NC, targeting 5′-TTCTCCGAACGTGTCACGT-3′) was used as the negative control.

GC cells (e.g., AGS, MKN45) were transfected with si-NGFR or si-NC (each at a final concentration of 100 nM) using Lipofectamine 3000 (Invitrogen, USA) according to the manufacturer’s protocol. Knockdown efficiency was confirmed by quantitative real-time PCR (RT-qPCR) and Western blot analysis 48 h post-transfection.

### RNA extraction and quantitative real-time polymerase chain reaction (RT-qPCR)

Total RNA was isolated from GC cells and tissues using Trizol Reagent (Invitrogen, Waltham, MA, USA). The RNA was then converted into cDNA using the Evo M-MLV RT Premix for qPCR Kit (Accurate Biology, Cat. No.: AG11706, China) according to the manufacturer’s instructions. RT-qPCR was performed using the SYBR Green Premix Pro Taq HS qPCR Kit (Accurate Biology, Cat. No.: AG11718, China) on a Roche LightCycler 480 Ⅱ system. Relative quantification was conducted via the 2^−ΔΔCt^ method to calculate fold changes, after normalization to the internal control glyceraldehyde-3-phosphate dehydrogenase (GAPDH). The primers used in this study are listed in Supplementary Table S2.

### Immunohistochemistry (IHC)

According to the manufacturer’s recommendations, IHC assays were performed using an IHC kit (ZSGB Bio, China). Paraffin-embedded tissue sections were incubated with primary antibodies: anti-PHOX1 (1:500; Abcam, Cat. No.: ab211292); anti-NGFR (1:500; Bioss Antibodies, Cat. No.: bs-7122R); anti-p-ERK (1:500; Cell Signaling Technology, Cat. No.: 4370S, USA); and anti-Ki67 (1:10,000; Huabio Technology, Cat. No.: HA721115, China) at 4 °C overnight. The IHC staining intensity was graded on a four-point scale: 0 indicating no staining; 1 representing faint brown; 2 corresponding to brown; and 3 denoting intense dark brown. The extent of staining was graded as: 0 for less than 5% of the area; 1 for 5% to 25%; 2 for 26% to 50%; 3 for 51% to 75%; and 4 for 76% to 100%. The overall staining score for paraffin-embedded tissues was determined using a quantitative assessment method, whereby the staining intensity grade was multiplied by the proportion of positively stained cells. Two independent pathologists who were unaware of the slide information performed IHC scoring, and the mean score of the two pathologists was adopted for each slide.

### Methylation-specific PCR (MSP) and unmethylation-specific PCR (USP)

To investigate the methylation status of GC tissues and cell lines, genomic DNA was extracted from these samples using a DNA Extraction Kit (Tiangen Biotech, Cat. No.: DP304, China). According to the manufacturer’s protocol, 500 ng of genomic DNA was treated with sodium bisulfite for bisulfite conversion using the EZ DNA Methylation Kit (Zymo Research, Cat. No.: D5001, USA). MSP was performed in a 50 μl reaction volume using the PerfectStart Taq DNA Polymerase Kit (TransGen Biotech, Cat. No.: AP401, Beijing, China). MSP and unmethylated-specific PCR (USP) products were separated by electrophoresis on 2% agarose gels stained with GoldView nucleic acid gel stain (Accurate Biology, Cat. No.: AG11915, China). Primers were designed using MethPrimer 2.0 (http://www.urogene.org/methprimer) and are listed in Supplementary Table S4.

### Methylation-specific quantitative real-time PCR (qMSP) and unmethylation-specific quantitative real-time PCR (qUSP)

To quantitatively determine the methylation status of the PHOX1 gene, RT-qPCR was performed on GC tissue DNA samples following bisulfite conversion. The primers are listed in Supplementary Table S4. Amplification was performed on a Roche LightCycler 480 II system with the following thermal cycling conditions: initial denaturation at 95 °C for 10 min, followed by 40 cycles of denaturation at 95 °C for 15 s and annealing and extension at 60 °C for 1 min. The *β-actin gene*, which maintains stable methylation status across different tissue samples, was used as the internal reference gene to normalize input DNA quantity. The *β-actin* primers (forward: 5′-GGGTGGTGATGGAGGAGGTT-3′; reverse: 5′-TAACCACCACCCAACACACAAT-3′) target a CpG-poor region in the genome to avoid methylation bias [37]. Each sample was analyzed in triplicate, and the relative methylation and unmethylation levels of the PHOX1 gene were calculated using the 2^−ΔΔCt^ method, where ΔCt represents the difference between the threshold cycle (Ct) values of PHOX1 and β-actin, and ΔΔCt is the normalized ΔCt value relative to the control group. The final results were presented as Mean ± standard deviation (SD) of three independent experiments.

### Bisulfite sequencing polymerase chain reaction (BSP)

Genomic DNA extraction and subsequent sodium bisulfite modification were performed following the BSP protocol. Primers for detecting methylation within the PHOX1 CpG island were designed using MethPrimer 2.0 software, with sequences listed in Supplementary Table S4. Next, bisulfite-modified DNA fragments were amplified following the manufacturer’s guidelines for the PerfectStart Taq DNA Polymerase Kit (TransGen Biotech, Cat. No.: AP401, China). Amplified fragments were subsequently purified using a DNA Gel Extraction Kit (TransGen Biotech, Cat. No.: DP209, Beijing, China). The PCR amplicons were then ligated into a T-vector and transformed into DH5α-competent *Escherichia coli* (*E. coli*) cells. Ten clones per sample were selected and cultured in Luria-Bertani (LB) medium on a shaker (200 rpm, 37 °C) overnight. Plasmid DNA was extracted using a TIANprep Plasmid Mini Kit (Tiangen Biotech, Cat. No.: DP118, China) and sequenced by Applied Biosystems (Waltham, MA, USA) using M13 primers.

### Western blot (WB) assay

To detect protein expression, proteins were first separated by sodium dodecyl sulfate-polyacrylamide gel electrophoresis (SDS-PAGE) and then transferred onto polyvinylidene fluoride (PVDF) membranes for subsequent immunodetection. For immunoblotting, membranes were incubated overnight (8–12 h) at 4 °C with specific primary antibodies, with details listed in Supplementary Table S5. After primary antibody incubation, membranes were washed three times (10 min each) with Tris-buffered saline containing 0.1% Tween-20 (TBST) to remove unbound primary antibodies. Subsequently, membranes were probed with species-specific horseradish peroxidase (HRP)-conjugated secondary antibodies—including Goat Anti-Mouse IgG H&L/HRP (diluted 1:10,000; Cat. No.: bs-40296G-HRP; Bioss Antibodies, China) and Goat Anti-Rabbit IgG H&L/HRP (diluted 1:10,000; Cat. No.: bs-0295G-HRP; Bioss Antibodies, China)—for 1 h at room temperature (22–25 °C) with gentle agitation. Finally, protein bands were visualized using Super Electrochemiluminescence (ECL) Detection Reagent (Yeasen Biotechnology, Cat. No.: 36208ES76, China) and a BLT Photon Technology Imager.

### 5-ethynyl-2-deoxyuridine (EDU) assay

To assess the proliferation rate of GC cells, cells were seeded in a 48-well plate (density optimized for experimental conditions) and supplemented with 200 μl of culture medium containing 10 μM EdU per well. Cells were incubated for 60 min in a humidified 37 °C, 5% CO₂ incubator to ensure sufficient EdU incorporation. After incubation, cells were fixed with freshly prepared 4% paraformaldehyde (PFA) dissolved in phosphate-buffered saline (PBS) for 30 min at room temperature—this step preserves cellular morphology and maintains antigen integrity for subsequent staining. Post-fixation, cells were permeabilized with 0.5% Triton ×-100 in PBS for 30 min to facilitate reagent penetration into the cytoplasm and nucleus. Cellular nuclei were then counterstained with Hoechst 33342 (a commonly used nuclear dye) for 10–15 min at room temperature to enable nuclear visualization and morphological assessment. For quantitative analysis of proliferation, the BeyoClick™ EdU-594 Kit (Beyotime Biotechnology, Cat. No.: C0078S, China) was used according to the manufacturer’s instructions. Five randomly selected fields of view were imaged per group using a Leica fluorescence microscope (Leica Microsystems, Wetzlar, Germany) at ×200 magnification; images were captured under consistent exposure parameters to ensure data comparability.

### Colony formation, transwell, and invasion assays

Cells were first seeded in 6-well culture plates at a density of 500 viable cells per well (low density), ensuring sufficient space for the formation of individual colonies. Cultures were maintained in complete growth medium (as detailed above) under standard incubation conditions (37 °C, 5% CO₂, 95% relative humidity) for 14 days to facilitate robust colony formation and expansion. After the incubation period, colonies were fixed with ice-cold methanol for 15 min at room temperature and subsequently stained with hematoxylin for 5 min to visualize colony morphology. For quantitative analysis, only well-defined colonies containing ≥50 cells were counted (to exclude small, fragmented cell clusters). To ensure experimental reproducibility and statistical robustness, the assay was performed with three independent biological replicates, each conducted in technical triplicate.

For cell transwell migration and invasion assays, the following procedures were performed: For migration assays, 1 × 10⁵ cells in serum-free medium were seeded into the upper chamber of Transwell inserts (8-μm pore size; Corning, USA). For invasion assays, inserts were pre-coated with Matrigel (Corning, USA) at 37 °C for 30 min to form a basement membrane-like barrier before seeding 1 × 10⁵ cells. The lower chamber was filled with complete medium containing 10% FBS as a chemoattractant. Cells were incubated under standard conditions (37 °C, 5% CO₂) for 24–72 h, with the duration adjusted based on cell type-specific migratory/invasive capacity. After incubation, non-migrated/non-invaded cells on the upper membrane surface were gently removed with a cotton swab. Migrated/invaded cells on the lower surface were fixed with freshly prepared 4% PFA for 15 min at room temperature and stained with hematoxylin for 5 min. Five randomly selected fields per insert were imaged using a light microscope, and cells were counted manually for quantitative analysis.

### Luciferase reporter assays

To investigate the regulatory mechanism of PHOX1 on NGFR expression, we first predicted high-probability PHOX1 binding sites in the *NGFR* promoter using the JASPAR database (http://jaspar.genereg.net), a comprehensive repository of transcription factor binding profiles. We then amplified the *NGFR* promoter fragment containing three candidate PHOX1 binding sites and subcloned it into the PGL3 luciferase reporter vector to generate the wild-type (WT) construct. Mutant constructs (Mut1-3) were further generated by introducing site-directed mutations at each of these three binding sites.

For luciferase reporter assays, AGS and MKN45 cells were co-transfected with the PHOX1 overexpression plasmid, PGL3 vectors (WT or Mut1-3), and pTK-Renilla luciferase vector (as an internal control) using Lipofectamine® 3000 (Thermo Fisher Scientific, USA). Luciferase activity was measured 48 h post-transfection using the Dual-Luciferase Reporter Assay System (TransDetect® Bright-Luc Firefly Luciferase Reporter Assay Kit, TransGen Biotech, China). Luminescence intensities of firefly luciferase (from PGL3) and Renilla luciferase (from pTK) were quantified using a microplate reader. Relative luciferase activity was calculated as the ratio of firefly to Renilla luciferase activity to normalize for variations in transfection efficiency and cell viability. For intergroup comparison, the relative activity of the WT reporter co-transfected with empty vector (control group) was set as 1.0. Activities of other groups—including WT with PHOX1 overexpression, and Mut1-3 constructs with PHOX1 overexpression—were expressed as fold changes relative to this control.

### Chromatin immunoprecipitation (ChIP)-qPCR

The ChIP assay was performed using the EZ-Magna ChIP® A/G Chromatin Immunoprecipitation Kit (Sigma-Aldrich, Cat. No.: 17-10086, USA). First, the cells were cross-linked with formaldehyde and then disrupted using lysis buffer. Next, the DNA was subjected to sonication on ice with a power setting of 40–60% amplitude (10–15 cycles of 30 s ON/30 s OFF) until DNA fragment sizes between 200 and 1000 base pairs were obtained. An anti-FLAG antibody (1:100 dilution; Proteintech, China) and normal rabbit IgG (as a negative control) were used to precipitate protein-DNA complexes, with rotation overnight at 4 °C. Adhering to the manufacturer’s protocol, the subsequent steps were performed. Following elution from the ChIP complexes, the DNA, along with 1% of the initial input DNA, was treated for reverse cross-linking at 65 °C overnight. Subsequently, the DNA samples were subjected to qPCR to validate potential PHOX1-binding sites within the *NGFR* promoter using the corresponding primers (Supplementary Table S3).

### RNA sequencing

Total RNA was extracted from AGS.NC (control) and AGS.PHOX1 (PHOX1-overexpressing) GC cells using TRIzol reagent (Invitrogen), followed by DNase I treatment to remove genomic DNA contamination. RNA quality and concentration were assessed by NanoDrop 2000 (OD260/280 ratio 1.8–2.0) and Agilent Bioanalyzer (RIN ≥ 8.0). mRNA was purified using oligo(dT) magnetic beads, and cDNA libraries were constructed with the NEBNext Ultra RNA Library Prep Kit for Illumina. Sequencing was performed on an Illumina HiSeq platform with 150-bp paired-end reads. Raw reads were filtered by FastQC, and clean reads were aligned to the human genome (GRCh38) using HISAT2. Gene expression was quantified by StringTie, and differentially expressed genes (DEGs) were identified via DESeq2 with criteria of |log2FC| ≥ 2 and adjusted *P* < 0.05. Biological replicates (*n* = 3) were included for each group to ensure reproducibility.

### Subcutaneous and orthotopic xenograft tumor mouse model

To assess the tumorigenic potential of GC cells, subcutaneous xenograft models were established in 4-week-old female BALB/c nude mice (nu/nu) purchased from the Guangdong Medical Laboratory Animal Center (Guangzhou, China). After a 1-week acclimation period in a specific pathogen-free-grade animal facility, mice were stratified by body weight to ensure baseline homogeneity, then randomly assigned to experimental groups via computer-generated randomization to minimize selection bias. Exponentially growing GC cells were collected and resuspended; 100 μl of cell suspension (containing 1 × 10⁶ cells, i.e., 1 × 10⁶ cells/100 μl) was injected subcutaneously into the dorsal flank of each mouse (*n* = 5 per group). Post-injection, subcutaneous tumors were monitored for 20 days: tumor length (longest diameter) and width (shortest diameter) were measured, and tumor volume was calculated using the standard formula for xenograft volume: tumor volume (mm³) = (length × width²)/2. Exclusion criteria: Mice that died of injection-related infection prior to the experimental endpoint were excluded from analysis. Tumor volume measurements were performed by investigators masked to group assignments (cages and sample slides were labeled only with codes); masking was lifted post-final data analysis to avoid measurement bias. Twenty days after cell inoculation, mice were euthanized via cervical dislocation (in accordance with institutional animal ethics guidelines). Xenograft tumors were harvested to determine tumor weight and reconfirm length/width; tumors were then fixed in 4% paraformaldehyde (4% PFA in PBS) for 24 h at 4 °C, followed by paraffin embedding for subsequent histological analysis.

Orthotopic GC models were established in 4-week-old female BALB/c nude mice. The methodology for establishing orthotopic GC models followed the procedures detailed in a previously published study [38]. Briefly, anesthesia was induced in the mice using ketamine at a dosage of 70 mg/kg body weight, and a minor abdominal incision was subsequently made. Next, the stomachs were exposed, and 100 μl of cell suspension containing 1 × 10⁶ cells was injected into the muscular layer of the stomachs. Subsequently, the mice were injected with penicillin-streptomycin (100 U/ml penicillin, 100 μg/ml streptomycin) to prevent infection, and the abdominal incision was closed with sutures. The mices were monitored for two months and euthanized when signs of cachexia appeared. Tumor tissues were harvested and fixed for subsequent analyses.

### 5-aza-2′-deoxycytidine (5-Aza) treatment

Cells in the exponential growth phase were plated in 60-mm cell dishes and incubated overnight to allow the cells to adhere and reach 30–50% confluency. The cells were then incubated with 5 μM 5-azacytidine (5-aza; Cat. No.: 189825, Sigma-Aldrich, USA; dissolved in DMSO) in a 37 °C, 5% CO₂ incubator for 72 h, with daily replacement of the culture medium. Following treatment, the cells were harvested to detect PHOX1 mRNA and protein levels.

### ERK inhibitor (SCH772984) treatment

For ERK inhibitor (SCH772984) treatment, GC cells in the exponential growth phase were plated in 60-mm cell culture dishes at a suitable density to allow for adhesion overnight. The cells were then treated with 5 μM SCH772984 (Cat. No.: HY-50846, MedChemExpress, USA; dissolved in DMSO) for 24 h. Control cells were treated with an equal volume of DMSO under identical culture conditions. All experiments were performed in triplicate (*n* = 3).

### Statistical analysis

Statistical analyses were performed using SPSS 22.0 and GraphPad Prism 9.0. Unless indicated otherwise in the figure legends, data were derived from at least three independent experiments, with each experiment including three technical replicates. Statistical tests were selected based on data type and experimental design: Comparisons between two groups: Two-tailed Student’s *t*-test (unpaired or paired, as appropriate for dependent/independent samples); Categorical data analysis: Chi-square test; Comparisons among three or more groups: One-way or two-way analysis of variance (ANOVA). Prior to performing parametric tests (Student’s *t*-test, one-way/two-way ANOVA), the Shapiro–Wilk test was used to assess the normality of continuous data. Normality testing confirmed that all continuous data conformed to a normal distribution, justifying the use of these parametric methods. For multiple comparisons following ANOVA, the Benjamini–Hochberg procedure was applied to control the false discovery rate and reduce Type I errors. Patient overall survival (OS) rates were estimated using KM survival analysis, and differences in survival curves between groups were compared using the log-rank test.

## Supplementary information

Bioinformatics R scripts

Supplementary Figures

Supplementary table

Original WB

## Data Availability

The transcriptomic data generated in this study have been deposited in the NCBI Sequence Read Archive (SRA) under the BioProject accession number PRJNA1293565 and Run accessions SRR34587201–SRR34587206, ensuring full reproducibility and transparency of the findings.
