# Supplementary material for: Hypomethylation-mediated upregulation of PHOX1 promotes gastric cancer progression via transactivation of NGFR
Source: Cell Death Discov. 2025 Nov 28;11:548. doi: 10.1038/s41420-025-02811-3 (PMC12663246; doi:10.1038/s41420-025-02811-3)
Supplement: Supplementary file 1 — Bioinformatics R scripts [file 41420_2025_2811_MOESM1_ESM.docx]

# Version info: R 4.2.2, Biobase 2.58.0, GEOquery 2.66.0, limma 3.54.0

################################################################

# **Differential expression analysis with limma : GSE54129**

library(GEOquery)

library(limma)

library(umap)

# load series and platform data from GEO

gset <- getGEO("GSE54129", GSEMatrix =TRUE, AnnotGPL=TRUE)

if (length(gset) > 1) idx <- grep("GPL570", attr(gset, "names")) else idx <- 1

gset <- gset[[idx]]

# make proper column names to match toptable

fvarLabels(gset) <- make.names(fvarLabels(gset))

# group membership for all samples

gsms <- paste0("00000000000000000000011111111111111111111111111111",

"11111111111111111111111111111111111111111111111111",

"11111111111111111111111111111111")

sml <- strsplit(gsms, split="")[[1]]

# log2 transformation

ex <- exprs(gset)

qx <- as.numeric(quantile(ex, c(0., 0.25, 0.5, 0.75, 0.99, 1.0), na.rm=T))

LogC <- (qx[5] > 100) ||

(qx[6]-qx[1] > 50 && qx[2] > 0)

if (LogC) { ex[which(ex <= 0)] <- NaN

exprs(gset) <- log2(ex) }

# assign samples to groups and set up design matrix

gs <- factor(sml)

groups <- make.names(c("N","T"))

levels(gs) <- groups

gset$group <- gs

design <- model.matrix(~group + 0, gset)

colnames(design) <- levels(gs)

gset <- gset[complete.cases(exprs(gset)), ] # skip missing values

fit <- lmFit(gset, design) # fit linear model

# set up contrasts of interest and recalculate model coefficients

cts <- paste(groups[1], groups[2], sep="-")

cont.matrix <- makeContrasts(contrasts=cts, levels=design)

fit2 <- contrasts.fit(fit, cont.matrix)

# compute statistics and table of top significant genes

fit2 <- eBayes(fit2, 0.01)

tT <- topTable(fit2, adjust="fdr", sort.by="B", number=250)

tT <- subset(tT, select=c("ID","adj.P.Val","P.Value","t","B","logFC","GB_ACC","SPOT_ID","Gene.Symbol","Gene.symbol","Gene.title"))

write.table(tT, file=stdout(), row.names=F, sep="\t")

# Visualize and quality control test results.

# Build histogram of P-values for all genes. Normal test

# assumption is that most genes are not differentially expressed.

tT2 <- topTable(fit2, adjust="fdr", sort.by="B", number=Inf)

hist(tT2$adj.P.Val, col = "grey", border = "white", xlab = "P-adj",

ylab = "Number of genes", main = "P-adj value distribution")

# summarize test results as "up", "down" or "not expressed"

dT <- decideTests(fit2, adjust.method="fdr", p.value=0.05, lfc=0)

# Venn diagram of results

vennDiagram(dT, circle.col=palette())

# create Q-Q plot for t-statistic

t.good <- which(!is.na(fit2$F)) # filter out bad probes

qqt(fit2$t[t.good], fit2$df.total[t.good], main="Moderated t statistic")

# volcano plot (log P-value vs log fold change)

colnames(fit2) # list contrast names

ct <- 1 # choose contrast of interest

# Please note that the code provided to generate graphs serves as a guidance to

# the users. It does not replicate the exact GEO2R web display due to multitude

# of graphical options.

#

# The following will produce basic volcano plot using limma function:

volcanoplot(fit2, coef=ct, main=colnames(fit2)[ct], pch=20,

highlight=length(which(dT[,ct]!=0)), names=rep('+', nrow(fit2)))

# MD plot (log fold change vs mean log expression)

# highlight statistically significant (p-adj < 0.05) probes

plotMD(fit2, column=ct, status=dT[,ct], legend=F, pch=20, cex=1)

abline(h=0)

################################################################

# General expression data analysis

ex <- exprs(gset)

# box-and-whisker plot

dev.new(width=3+ncol(gset)/6, height=5)

ord <- order(gs) # order samples by group

palette(c("#1B9E77", "#7570B3", "#E7298A", "#E6AB02", "#D95F02",

"#66A61E", "#A6761D", "#B32424", "#B324B3", "#666666"))

par(mar=c(7,4,2,1))

title <- paste ("GSE54129", "/", annotation(gset), sep ="")

boxplot(ex[,ord], boxwex=0.6, notch=T, main=title, outline=FALSE, las=2, col=gs[ord])

legend("topleft", groups, fill=palette(), bty="n")

dev.off()

# expression value distribution

par(mar=c(4,4,2,1))

title <- paste ("GSE54129", "/", annotation(gset), " value distribution", sep ="")

plotDensities(ex, group=gs, main=title, legend ="topright")

# UMAP plot (dimensionality reduction)

ex <- na.omit(ex) # eliminate rows with NAs

ex <- ex[!duplicated(ex), ] # remove duplicates

ump <- umap(t(ex), n_neighbors = 15, random_state = 123)

par(mar=c(3,3,2,6), xpd=TRUE)

plot(ump$layout, main="UMAP plot, nbrs=15", xlab="", ylab="", col=gs, pch=20, cex=1.5)

legend("topright", inset=c(-0.15,0), legend=levels(gs), pch=20,

col=1:nlevels(gs), title="Group", pt.cex=1.5)

library("maptools") # point labels without overlaps

pointLabel(ump$layout, labels = rownames(ump$layout), method="SANN", cex=0.6)

# mean-variance trend, helps to see if precision weights are needed

plotSA(fit2, main="Mean variance trend, GSE54129")

# Version info: R 4.2.2, Biobase 2.58.0, GEOquery 2.66.0, limma 3.54.0

################################################################

# **Differential expression analysis with limma : GSE79973**

library(GEOquery)

library(limma)

library(umap)

# load series and platform data from GEO

gset <- getGEO("GSE79973", GSEMatrix =TRUE, AnnotGPL=TRUE)

if (length(gset) > 1) idx <- grep("GPL570", attr(gset, "names")) else idx <- 1

gset <- gset[[idx]]

# make proper column names to match toptable

fvarLabels(gset) <- make.names(fvarLabels(gset))

# group membership for all samples

gsms <- "10101010101010101010"

sml <- strsplit(gsms, split="")[[1]]

# log2 transformation

ex <- exprs(gset)

qx <- as.numeric(quantile(ex, c(0., 0.25, 0.5, 0.75, 0.99, 1.0), na.rm=T))

LogC <- (qx[5] > 100) ||

(qx[6]-qx[1] > 50 && qx[2] > 0)

if (LogC) { ex[which(ex <= 0)] <- NaN

exprs(gset) <- log2(ex) }

# assign samples to groups and set up design matrix

gs <- factor(sml)

groups <- make.names(c("N","T"))

levels(gs) <- groups

gset$group <- gs

design <- model.matrix(~group + 0, gset)

colnames(design) <- levels(gs)

gset <- gset[complete.cases(exprs(gset)), ] # skip missing values

fit <- lmFit(gset, design) # fit linear model

# set up contrasts of interest and recalculate model coefficients

cts <- paste(groups[1], groups[2], sep="-")

cont.matrix <- makeContrasts(contrasts=cts, levels=design)

fit2 <- contrasts.fit(fit, cont.matrix)

# compute statistics and table of top significant genes

fit2 <- eBayes(fit2, 0.01)

tT <- topTable(fit2, adjust="fdr", sort.by="B", number=250)

tT <- subset(tT, select=c("ID","adj.P.Val","P.Value","t","B","logFC","GB_ACC","SPOT_ID","Gene.Symbol","Gene.symbol","Gene.title"))

write.table(tT, file=stdout(), row.names=F, sep="\t")

# Visualize and quality control test results.

# Build histogram of P-values for all genes. Normal test

# assumption is that most genes are not differentially expressed.

tT2 <- topTable(fit2, adjust="fdr", sort.by="B", number=Inf)

hist(tT2$adj.P.Val, col = "grey", border = "white", xlab = "P-adj",

ylab = "Number of genes", main = "P-adj value distribution")

# summarize test results as "up", "down" or "not expressed"

dT <- decideTests(fit2, adjust.method="fdr", p.value=0.05, lfc=0)

# Venn diagram of results

vennDiagram(dT, circle.col=palette())

# create Q-Q plot for t-statistic

t.good <- which(!is.na(fit2$F)) # filter out bad probes

qqt(fit2$t[t.good], fit2$df.total[t.good], main="Moderated t statistic")

# volcano plot (log P-value vs log fold change)

colnames(fit2) # list contrast names

ct <- 1 # choose contrast of interest

# Please note that the code provided to generate graphs serves as a guidance to

# the users. It does not replicate the exact GEO2R web display due to multitude

# of graphical options.

#

# The following will produce basic volcano plot using limma function:

volcanoplot(fit2, coef=ct, main=colnames(fit2)[ct], pch=20,

highlight=length(which(dT[,ct]!=0)), names=rep('+', nrow(fit2)))

# MD plot (log fold change vs mean log expression)

# highlight statistically significant (p-adj < 0.05) probes

plotMD(fit2, column=ct, status=dT[,ct], legend=F, pch=20, cex=1)

abline(h=0)

################################################################

# General expression data analysis

ex <- exprs(gset)

# box-and-whisker plot

ord <- order(gs) # order samples by group

palette(c("#1B9E77", "#7570B3", "#E7298A", "#E6AB02", "#D95F02",

"#66A61E", "#A6761D", "#B32424", "#B324B3", "#666666"))

par(mar=c(7,4,2,1))

title <- paste ("GSE79973", "/", annotation(gset), sep ="")

boxplot(ex[,ord], boxwex=0.6, notch=T, main=title, outline=FALSE, las=2, col=gs[ord])

legend("topleft", groups, fill=palette(), bty="n")

# expression value distribution

par(mar=c(4,4,2,1))

title <- paste ("GSE79973", "/", annotation(gset), " value distribution", sep ="")

plotDensities(ex, group=gs, main=title, legend ="topright")

# UMAP plot (dimensionality reduction)

ex <- na.omit(ex) # eliminate rows with NAs

ex <- ex[!duplicated(ex), ] # remove duplicates

ump <- umap(t(ex), n_neighbors = 9, random_state = 123)

par(mar=c(3,3,2,6), xpd=TRUE)

plot(ump$layout, main="UMAP plot, nbrs=9", xlab="", ylab="", col=gs, pch=20, cex=1.5)

legend("topright", inset=c(-0.15,0), legend=levels(gs), pch=20,

col=1:nlevels(gs), title="Group", pt.cex=1.5)

library("maptools") # point labels without overlaps

pointLabel(ump$layout, labels = rownames(ump$layout), method="SANN", cex=0.6)

# mean-variance trend, helps to see if precision weights are needed

plotSA(fit2, main="Mean variance trend, GSE79973")

# Version info: R 4.2.2, Biobase 2.58.0, GEOquery 2.66.0, limma 3.54.0

################################################################

# **Differential expression analysis with limma : GS118916**

library(GEOquery)

library(limma)

library(umap)

# load series and platform data from GEO

gset <- getGEO("GSE118916", GSEMatrix =TRUE, AnnotGPL=FALSE)

if (length(gset) > 1) idx <- grep("GPL15207", attr(gset, "names")) else idx <- 1

gset <- gset[[idx]]

# make proper column names to match toptable

fvarLabels(gset) <- make.names(fvarLabels(gset))

# group membership for all samples

gsms <- "111111111111111000000000000000"

sml <- strsplit(gsms, split="")[[1]]

# log2 transformation

ex <- exprs(gset)

qx <- as.numeric(quantile(ex, c(0., 0.25, 0.5, 0.75, 0.99, 1.0), na.rm=T))

LogC <- (qx[5] > 100) ||

(qx[6]-qx[1] > 50 && qx[2] > 0)

if (LogC) { ex[which(ex <= 0)] <- NaN

exprs(gset) <- log2(ex) }

# assign samples to groups and set up design matrix

gs <- factor(sml)

groups <- make.names(c("N","T"))

levels(gs) <- groups

gset$group <- gs

design <- model.matrix(~group + 0, gset)

colnames(design) <- levels(gs)

gset <- gset[complete.cases(exprs(gset)), ] # skip missing values

fit <- lmFit(gset, design) # fit linear model

# set up contrasts of interest and recalculate model coefficients

cts <- paste(groups[1], groups[2], sep="-")

cont.matrix <- makeContrasts(contrasts=cts, levels=design)

fit2 <- contrasts.fit(fit, cont.matrix)

# compute statistics and table of top significant genes

fit2 <- eBayes(fit2, 0.01)

tT <- topTable(fit2, adjust="fdr", sort.by="B", number=250)

tT <- subset(tT, select=c("ID","adj.P.Val","P.Value","t","B","logFC","GB_ACC","GI","Gene.Symbol","SPOT_ID"))

write.table(tT, file=stdout(), row.names=F, sep="\t")

# Visualize and quality control test results.

# Build histogram of P-values for all genes. Normal test

# assumption is that most genes are not differentially expressed.

tT2 <- topTable(fit2, adjust="fdr", sort.by="B", number=Inf)

hist(tT2$adj.P.Val, col = "grey", border = "white", xlab = "P-adj",

ylab = "Number of genes", main = "P-adj value distribution")

# summarize test results as "up", "down" or "not expressed"

dT <- decideTests(fit2, adjust.method="fdr", p.value=0.05, lfc=0)

# Venn diagram of results

vennDiagram(dT, circle.col=palette())

# create Q-Q plot for t-statistic

t.good <- which(!is.na(fit2$F)) # filter out bad probes

qqt(fit2$t[t.good], fit2$df.total[t.good], main="Moderated t statistic")

# volcano plot (log P-value vs log fold change)

colnames(fit2) # list contrast names

ct <- 1 # choose contrast of interest

# Please note that the code provided to generate graphs serves as a guidance to

# the users. It does not replicate the exact GEO2R web display due to multitude

# of graphical options.

#

# The following will produce basic volcano plot using limma function:

volcanoplot(fit2, coef=ct, main=colnames(fit2)[ct], pch=20,

highlight=length(which(dT[,ct]!=0)), names=rep('+', nrow(fit2)))

# MD plot (log fold change vs mean log expression)

# highlight statistically significant (p-adj < 0.05) probes

plotMD(fit2, column=ct, status=dT[,ct], legend=F, pch=20, cex=1)

abline(h=0)

################################################################

# General expression data analysis

ex <- exprs(gset)

# box-and-whisker plot

dev.new(width=3+ncol(gset)/6, height=5)

ord <- order(gs) # order samples by group

palette(c("#1B9E77", "#7570B3", "#E7298A", "#E6AB02", "#D95F02",

"#66A61E", "#A6761D", "#B32424", "#B324B3", "#666666"))

par(mar=c(7,4,2,1))

title <- paste ("GSE118916", "/", annotation(gset), sep ="")

boxplot(ex[,ord], boxwex=0.6, notch=T, main=title, outline=FALSE, las=2, col=gs[ord])

legend("topleft", groups, fill=palette(), bty="n")

dev.off()

# expression value distribution

par(mar=c(4,4,2,1))

title <- paste ("GSE118916", "/", annotation(gset), " value distribution", sep ="")

plotDensities(ex, group=gs, main=title, legend ="topright")

# UMAP plot (dimensionality reduction)

ex <- na.omit(ex) # eliminate rows with NAs

ex <- ex[!duplicated(ex), ] # remove duplicates

ump <- umap(t(ex), n_neighbors = 13, random_state = 123)

par(mar=c(3,3,2,6), xpd=TRUE)

plot(ump$layout, main="UMAP plot, nbrs=13", xlab="", ylab="", col=gs, pch=20, cex=1.5)

legend("topright", inset=c(-0.15,0), legend=levels(gs), pch=20,

col=1:nlevels(gs), title="Group", pt.cex=1.5)

library("maptools") # point labels without overlaps

pointLabel(ump$layout, labels = rownames(ump$layout), method="SANN", cex=0.6)

# mean-variance trend, helps to see if precision weights are needed

plotSA(fit2, main="Mean variance trend, GSE118916")

# Version info: R 4.2.2, Biobase 2.58.0, GEOquery 2.66.0, limma 3.54.0

################################################################

# **Differential expression analysis with limma: GSE19826**

library(GEOquery)

library(limma)

library(umap)

# load series and platform data from GEO

gset <- getGEO("GSE19826", GSEMatrix =TRUE, AnnotGPL=TRUE)

if (length(gset) > 1) idx <- grep("GPL570", attr(gset, "names")) else idx <- 1

gset <- gset[[idx]]

# make proper column names to match toptable

fvarLabels(gset) <- make.names(fvarLabels(gset))

# group membership for all samples

gsms <- "010101010101010101010101000"

sml <- strsplit(gsms, split="")[[1]]

# log2 transformation

ex <- exprs(gset)

qx <- as.numeric(quantile(ex, c(0., 0.25, 0.5, 0.75, 0.99, 1.0), na.rm=T))

LogC <- (qx[5] > 100) ||

(qx[6]-qx[1] > 50 && qx[2] > 0)

if (LogC) { ex[which(ex <= 0)] <- NaN

exprs(gset) <- log2(ex) }

# assign samples to groups and set up design matrix

gs <- factor(sml)

groups <- make.names(c("N","T"))

levels(gs) <- groups

gset$group <- gs

design <- model.matrix(~group + 0, gset)

colnames(design) <- levels(gs)

gset <- gset[complete.cases(exprs(gset)), ] # skip missing values

fit <- lmFit(gset, design) # fit linear model

# set up contrasts of interest and recalculate model coefficients

cts <- paste(groups[1], groups[2], sep="-")

cont.matrix <- makeContrasts(contrasts=cts, levels=design)

fit2 <- contrasts.fit(fit, cont.matrix)

# compute statistics and table of top significant genes

fit2 <- eBayes(fit2, 0.01)

tT <- topTable(fit2, adjust="fdr", sort.by="B", number=250)

tT <- subset(tT, select=c("ID","adj.P.Val","P.Value","t","B","logFC","GB_ACC","SPOT_ID","Gene.Symbol","Gene.symbol","Gene.title"))

write.table(tT, file=stdout(), row.names=F, sep="\t")

# Visualize and quality control test results.

# Build histogram of P-values for all genes. Normal test

# assumption is that most genes are not differentially expressed.

tT2 <- topTable(fit2, adjust="fdr", sort.by="B", number=Inf)

hist(tT2$adj.P.Val, col = "grey", border = "white", xlab = "P-adj",

ylab = "Number of genes", main = "P-adj value distribution")

# summarize test results as "up", "down" or "not expressed"

dT <- decideTests(fit2, adjust.method="fdr", p.value=0.05, lfc=0)

# Venn diagram of results

vennDiagram(dT, circle.col=palette())

# create Q-Q plot for t-statistic

t.good <- which(!is.na(fit2$F)) # filter out bad probes

qqt(fit2$t[t.good], fit2$df.total[t.good], main="Moderated t statistic")

# volcano plot (log P-value vs log fold change)

colnames(fit2) # list contrast names

ct <- 1 # choose contrast of interest

# Please note that the code provided to generate graphs serves as a guidance to

# the users. It does not replicate the exact GEO2R web display due to multitude

# of graphical options.

#

# The following will produce basic volcano plot using limma function:

volcanoplot(fit2, coef=ct, main=colnames(fit2)[ct], pch=20,

highlight=length(which(dT[,ct]!=0)), names=rep('+', nrow(fit2)))

# MD plot (log fold change vs mean log expression)

# highlight statistically significant (p-adj < 0.05) probes

plotMD(fit2, column=ct, status=dT[,ct], legend=F, pch=20, cex=1)

abline(h=0)

################################################################

# General expression data analysis

ex <- exprs(gset)

# box-and-whisker plot

ord <- order(gs) # order samples by group

palette(c("#1B9E77", "#7570B3", "#E7298A", "#E6AB02", "#D95F02",

"#66A61E", "#A6761D", "#B32424", "#B324B3", "#666666"))

par(mar=c(7,4,2,1))

title <- paste ("GSE19826", "/", annotation(gset), sep ="")

boxplot(ex[,ord], boxwex=0.6, notch=T, main=title, outline=FALSE, las=2, col=gs[ord])

legend("topleft", groups, fill=palette(), bty="n")

# expression value distribution

par(mar=c(4,4,2,1))

title <- paste ("GSE19826", "/", annotation(gset), " value distribution", sep ="")

plotDensities(ex, group=gs, main=title, legend ="topright")

# UMAP plot (dimensionality reduction)

ex <- na.omit(ex) # eliminate rows with NAs

ex <- ex[!duplicated(ex), ] # remove duplicates

ump <- umap(t(ex), n_neighbors = 11, random_state = 123)

par(mar=c(3,3,2,6), xpd=TRUE)

plot(ump$layout, main="UMAP plot, nbrs=11", xlab="", ylab="", col=gs, pch=20, cex=1.5)

legend("topright", inset=c(-0.15,0), legend=levels(gs), pch=20,

col=1:nlevels(gs), title="Group", pt.cex=1.5)

library("maptools") # point labels without overlaps

pointLabel(ump$layout, labels = rownames(ump$layout), method="SANN", cex=0.6)

# mean-variance trend, helps to see if precision weights are needed

plotSA(fit2, main="Mean variance trend, GSE19826")
