# Supplementary material for: Hypomethylation-mediated upregulation of PHOX1 promotes gastric cancer progression via transactivation of NGFR
Source: Cell Death Discov. 2025 Nov 28;11:548. doi: 10.1038/s41420-025-02811-3 (PMC12663246; doi:10.1038/s41420-025-02811-3)
Supplement: Supplementary file 2 — Supplementary Figures [file 41420_2025_2811_MOESM2_ESM.docx]

**
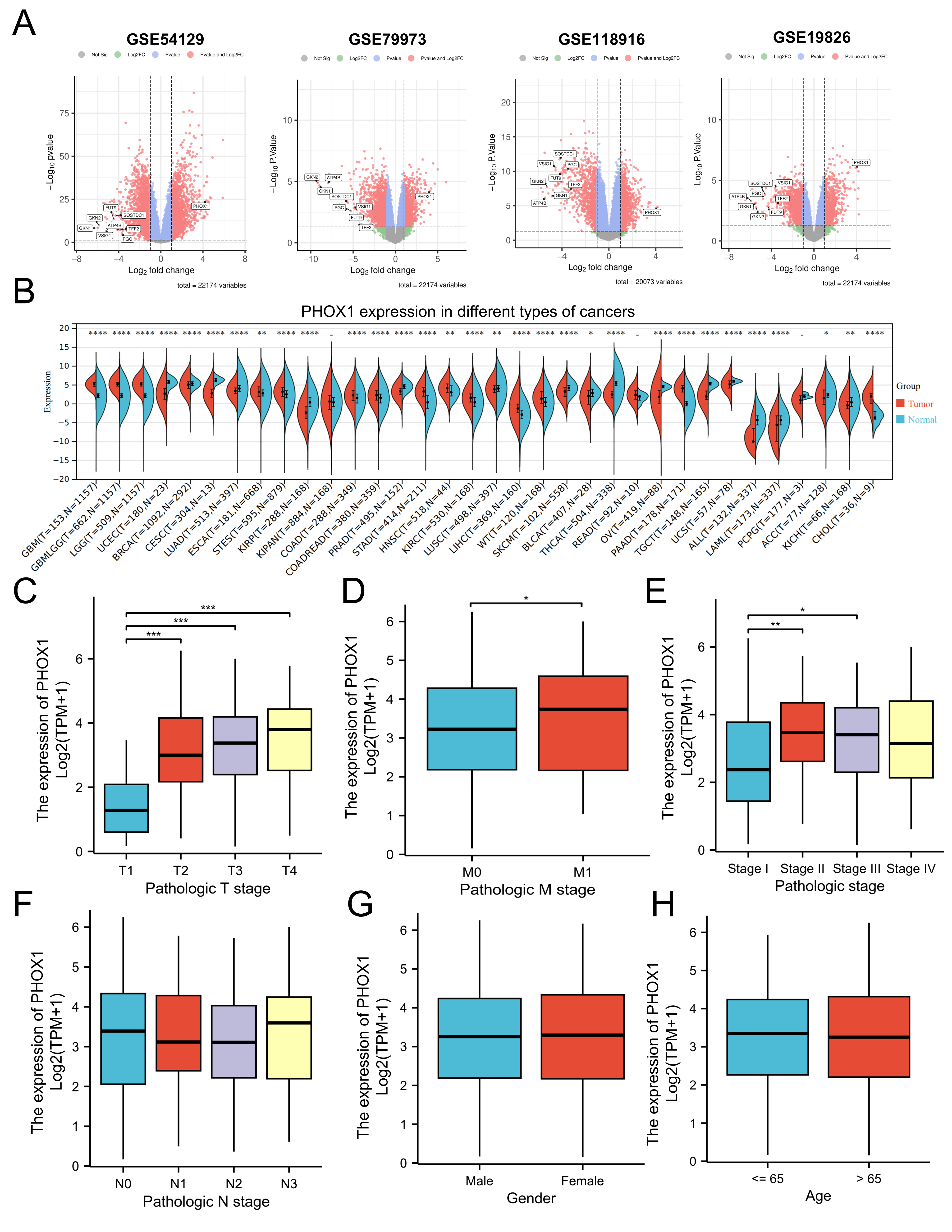
**

**Fig. S1** PHOX1 overexpression correlates with clinical metastasis in gastric cancer (GC).

**A.** Volcano plots showing all differentially expressed genes (DEGs) in each dataset. Red dots represent genes with |LogFC| ≥ 1 and P < 0.05; gray dots represent unchanged genes. **B.** Expression of PHOX1 across 34 cancer types from the TCGA and GTEx cohorts, analyzed using the SangerBox platform. **C.** Bar plot showing PHOX1 expression across different pathological T stages in the TCGA_STAD dataset. **D.** PHOX1 expression across different pathological M stages (M0 and M1) in the TCGA_STAD dataset. **E.** PHOX1 expression across different pathological stages (Stage I–IV) in the TCGA_STAD dataset. **F.** PHOX1 expression across different pathological N stages (N0, N1, N2, N3) in the TCGA_STAD dataset. **G.** PHOX1 expression across different genders (Male and Female) in the TCGA_STAD dataset. **H.** PHOX1 expression across different age groups (≤65 years and >65 years) in the TCGA_STAD dataset. * *P* <0.05, ** *P* <0.01, *** *P* < 0.001, **** *P* <0.0001.


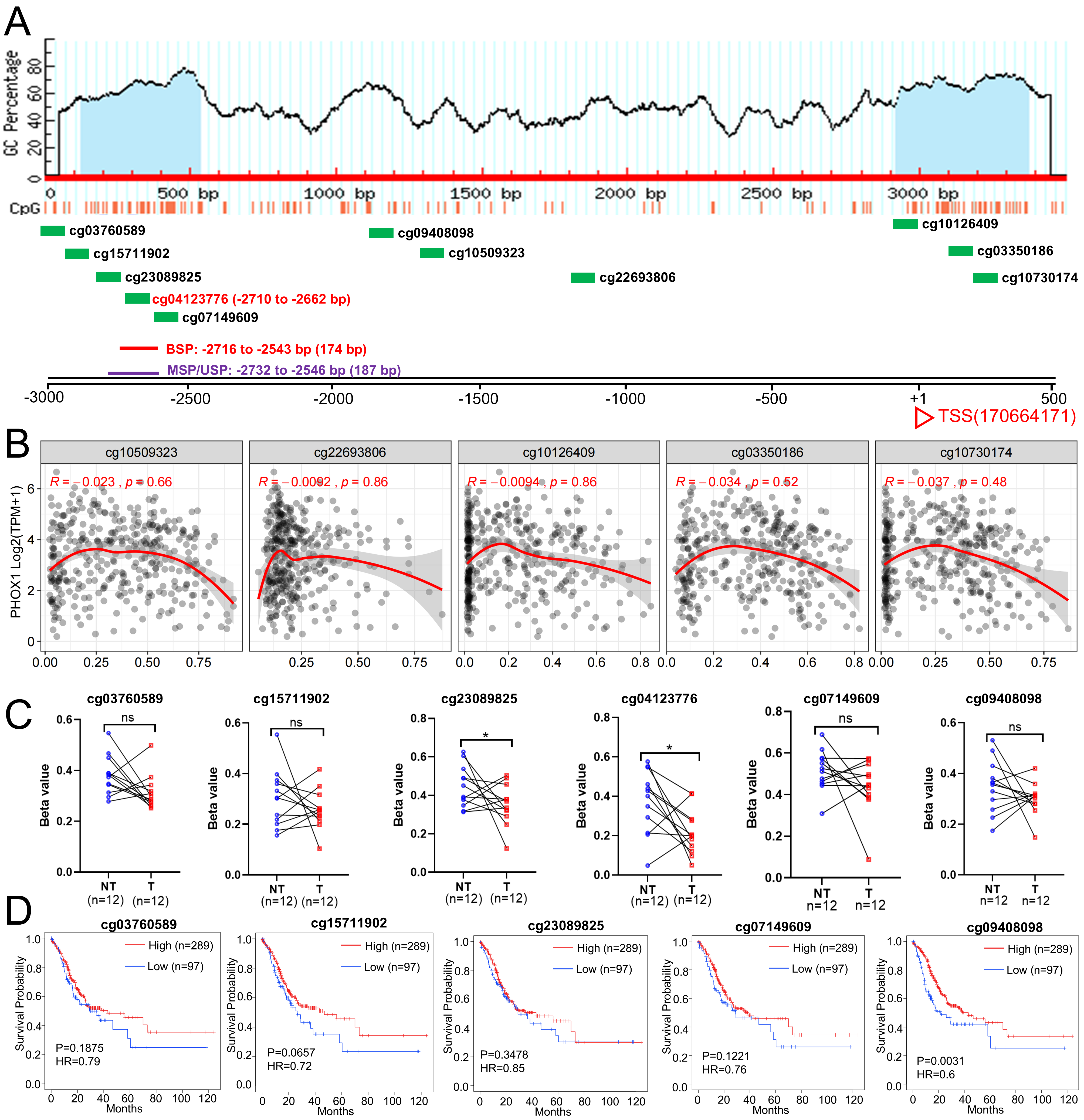


**Fig. S2** Promoter hypomethylation of PHOX1 is associated with poor prognosis in GC.

**A.** CpG methylation islands and primer product lengths in the *PHOX1* promoter, analyzed using MethPrimer. Input sequence: red region; CpG islands (CGIs): blue region. TSS: Transcription Start Site. **B.** Correlation analysis between PHOX1 expression and methylation of PHOX1-related CpG sites, using the SMART App. **C.** Methylation levels of six significant CpG sites (cg03760589, cg15711902, cg23089825, cg04123776, cg07149609, cg09408098) in PHOX1, measured in matched GC patient tissue pairs from the GSE164988 dataset. **D.** Survival analysis based on methylation status of CpG sites in the PHOX1 promoter, using the SMART platform. * *P* <0.05, ns: no significance.


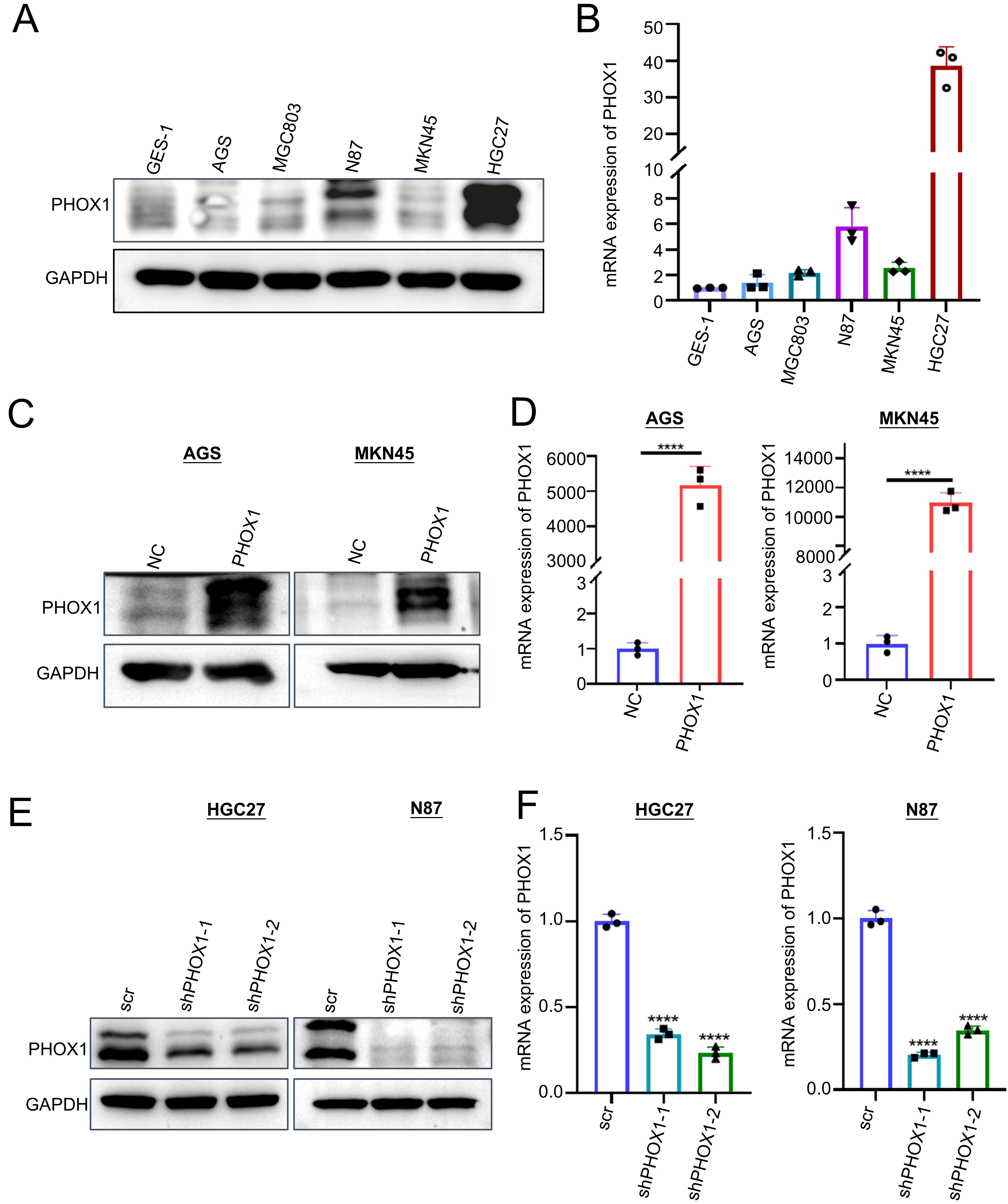


**Fig. S3** The construction of stable PHOX1-overexpressing and PHOX1-knockdown GC cell lines.

**A-B.** PHOX1 mRNA and protein expression in GES-1 cells and five GC cell lines. Data are presented as the mean ± SD (n ≥ 3). **C-F.** Western blot and RT-qPCR assays were performed to verify the successful construction of stable PHOX1-overexpressing and PHOX1-knockdown GC cell lines.


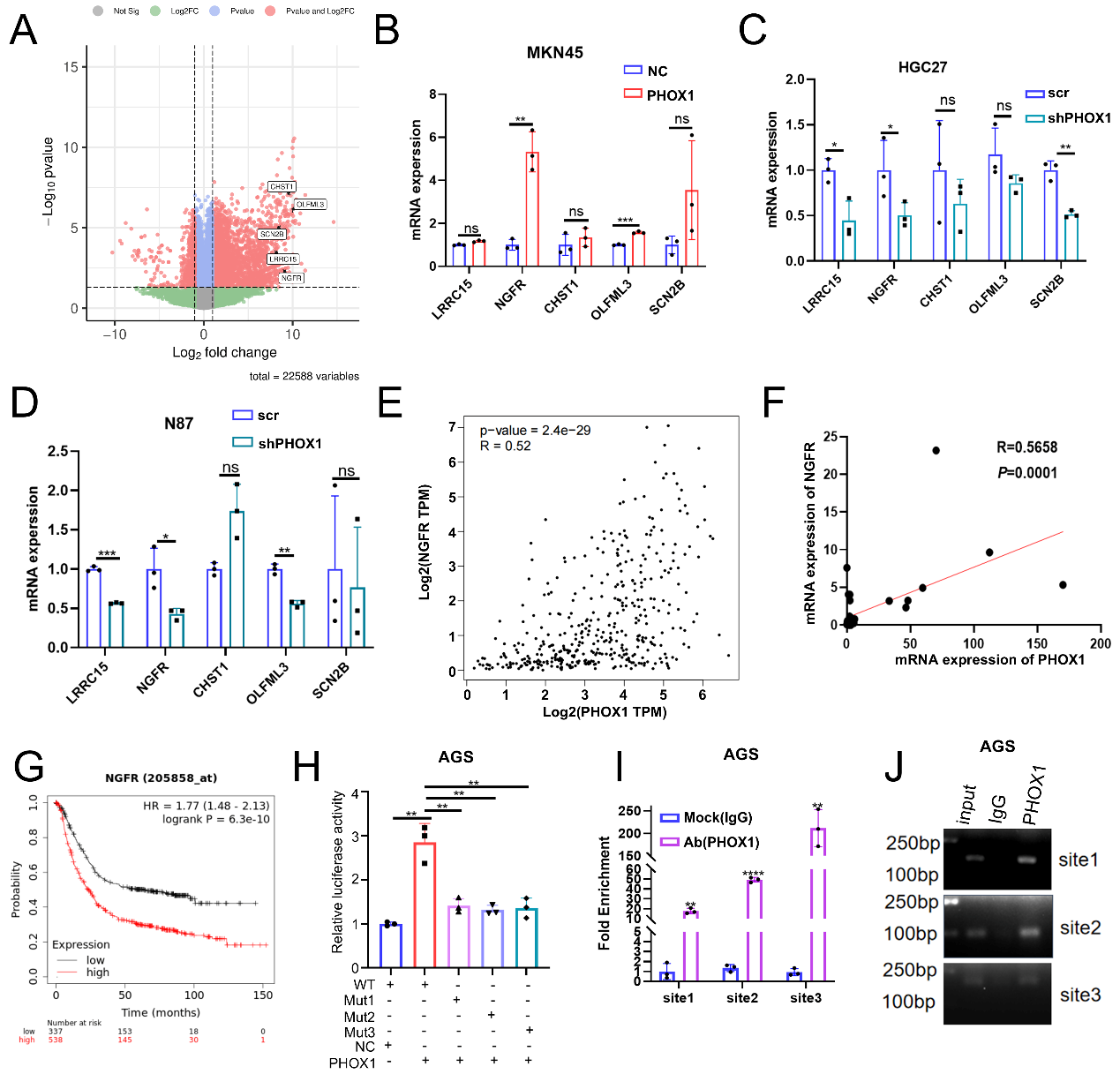


**Fig. S4** PHOX1 activates ERK1/2 signaling through NGFR upregulation

**A.** Volcano plot showing all differentially expressed genes (DEGs) in AGS cells with PHOX1 overexpression vs. empty vector control (NC). Red dots represent genes with |LogFC| ≥ 1 and P < 0.05; gray dots represent unchanged genes. **B-D.** Effect of PHOX1 overexpression on the expression of candidate DEGs in MKN45, HGC27, and N87 cells, assessed by RT-qPCR. **E.** Correlation between PHOX1 and NGFR expression levels in GC tissues, analyzed using the GEPIA2 platform. **F.** Correlation between PHOX1 and NGFR mRNA expression levels in 40 GC tissues, assessed by RT-qPCR. Pearson correlation analysis was used (n = 40). **G.** Survival analysis of GC patients based on NGFR expression, performed using the KM Plotter database. **H.** Luciferase reporter plasmids containing wild-type (WT) or mutant (Mut) NGFR promoter sequences were co-transfected with PHOX1-overexpressing plasmid or empty vector (NC) into GC cells, followed by luciferase reporter assays. **I.** ChIP-qPCR analysis of PHOX1 enrichment on the NGFR promoter in control vs. PHOX1-overexpressing GC cells. **J.** Agarose gel electrophoresis showing amplification of PHOX1-binding sites 1, 2, and 3 on the NGFR promoter following ChIP analysis. Molecular weight markers are indicated alongside the gels. * *P* <0.05, ** *P* <0.01, *** *P* < 0.001, ns: no significance.
