# Supplementary material for: Hypomethylation-mediated upregulation of PHOX1 promotes gastric cancer progression via transactivation of NGFR
Source: Cell Death Discov. 2025 Nov 28;11:548. doi: 10.1038/s41420-025-02811-3 (PMC12663246; doi:10.1038/s41420-025-02811-3)
Supplement: Supplementary file 3 — Supplementary table [file 41420_2025_2811_MOESM3_ESM.docx]

**Supplementary Table S1.** Association of PHOX1 expression with clinicopathological characteristics of GC patients

| **Clinicopathological**  **variables** | **PHOX1 expression**  **Low High** | | **Total** | **p-value** |
| --- | --- | --- | --- | --- |
| **Age(years)** |  |  |  |  |
| <60 | 6 | 17 | 23 | 0.533 |
| ≥60 | 17 | 34 | 51 |  |
| **Gender** |  |  |  |  |
| Male | 16 | 37 | 21 | 0.792 |
| Female | 7 | 14 | 53 |  |
| **Tumor depth** |  |  |  |  |
| T1 | 7 | 7 | 14 | 0.031 |
| T2 | 7 | 6 | 13 |  |
| T3 | 6 | 29 | 35 |  |
| T4 | 3 | 9 | 12 |  |
| **Lymph node** |  |  |  |  |
| N0 | 16 | 11 | 27 | 0.001 |
| N1 | 5 | 19 | 24 |  |
| N2 | 1 | 6 | 7 |  |
| N3 | 1 | 15 | 16 |  |
| **Metastasis** |  |  |  |  |
| M0 | 23 | 39 | 62 | 0.011 |
| M1 | 0 | 12 | 12 |  |
| **Histological grade** |  |  |  |  |
| Well | 5 | 28 | 33 | 0.029 |
| Moderate | 12 | 15 | 27 |  |
| Poor | 6 | 8 | 14 |  |
| **Stages** |  |  |  |  |
| I | 12 | 8 | 20 | 0.002 |
| II | 5 | 21 | 26 |  |
| III | 6 | 10 | 16 |  |
| IV | 0 | 12 | 12 |  |

**Supplementary Table S2.** Sequences of RT-qPCR primers

| **Gene** | **Forward primer** | **Reverse primer** | **Location** | **Product length** |
| --- | --- | --- | --- | --- |
| GAPDH | GGAGCGAGATCCCTCCAAAAT | GGCTGTTGTCATACTTCTCATGG | 108-304 bp | 197 bp |
| PHOX1 | CAGGCGGATGAGAACGTGG | AAAAGCATCAGGATAGTGTGTCC | 154-366 bp | 213 bp |
| NGFR | CCGTTGGATTACACGGTCCAC | TGAAGGCTATGTAGGCCACAA | 579-817 bp | 239 bp |
| NOVA2 | AAGGCGAATACTTCCTGAAGGT | TACTAGGCATACCCGCTCTGT | 89-255 bp | 167 bp |
| DACT3 | CCCAGCGTCGTCTGCTTTA | CGATTCGCTCTCCCCGTAAC | 1526-1650 bp | 125 bp |
| TMEM119 | CGGCCTATTACCCATCGTCC | CTGGGCTAACAAGAGAGACCC | 374-783 bp | 410 bp |
| CHST1 | ACCTGGCTCGGAACCCTAT | CGGTGCCGTATTTGTGCTTG | 911-1048 bp | 138 bp |
| SHANK1 | CGGACCTGCACCAGACAAAAT | GTTGGAACAGGCCATAGTTGAG | 239-364 bp | 126 bp |
| LRRC15 | TGCCCTAGCGAGTGTACCT | GATGTGCGTGTTGAGGATCTG | 73-195 bp | 123 bp |
| OLFML3 | GCGGGAGGTAGACTATCTGGA | CTTGAGAGATTGTGTAGCCACAG | 273-424 bp | 152 bp |
| FOXS1 | AGTGGCATCTACCGCTACATC | CACCTTGACAAAGCACTCGT | 124-237 bp | 114 bp |
| SCN2B | TGAACTGGACTTACCAGGAGTG | CTGGGGTTCCCTGAGAACT | 194-317 bp | 124 bp |
| CYTH4 | TTGCACGGTTCCTGTATAAAGG | GAGGTTGAGGTTGGCGAACT | 287-420 bp | 134 bp |

**Supplementary Table S3.** Sequences of ChIP-qPCR primers

| **Name** | **Forward primer** | **Reverse primer** | **Location** | **Product length** |
| --- | --- | --- | --- | --- |
| Site1 | ACATCTTAGAAGCAGTCCC | CTGGAAAGGACAGGTAGAC | 11-163 bp | 153 bp |
| Site2 | CCTGGACTTTGTGGGTTAC | CCAAGATCGGCTGAAGCT | 177-267 bp | 91 bp |
| Site3 | TTTCCGCTCACTGCAACC | CCTGTAATCCCAGCACTTT | 388-601 bp | 214 bp |

**Supplementary Table S4.** Sequences of RT-qPCR primers for MSP, USP and BSP

| **Name** | **Forward primer** | **Reverse primer** | **Location** | **Product length** |
| --- | --- | --- | --- | --- |
| Site1_M | GAGGTAGTTGAGTTGGAAATTTCGAC | CTACCAACGCCGCACTATACG | -2706~ -2533 bp | 187 bp |
| Site1_U | GGTAGTTGAGTTGGAAATTTTGATG | ACCCTACCAACACCACACTATACAC | -2732~ -2546 bp | 188 bp |
| Site1_B | GGAAAGTATTTATAAGTTTTTATTTTG | AAAACCCAAAACCCTACCAAC | -2730~ -2543 bp | 174 bp |

**Supplementary Table S5.** Antibodies information

| Antibody | Source | Catalog Number | Application |
| --- | --- | --- | --- |
| PHOX1 | Abclonal | A10237 | WB |
| NGFR | Bioss | bs-7122R | WB/IHC |
| ERK1/2 | CST | 4695T | WB |
| P-ERK1/2 | CST | 4370S | WB/IHC |
| JNK | CST | 9252T | WB |
| P-JNK | CST | 4668T | WB |
| P38 | CST | 8690T | WB |
| P-P38 | CST | 4511T | WB |
| GAPDH | Proteintech | 60004-1-Ig | WB |
| KI67 | Huabio | HA721115 | IHC |
| PHOX1 | Abcam | ab211292 | IHC |
| MMP1 | Proteintech | 10371-2-AP | WB |
| CCND1 | Proteintech | 26939-1-AP | WB |
| FOS | Abclonal | A24620 | WB |
| VEGFA | Proteintech | 66828-1-Ig | WB |

CST: Cell Signaling Technology; WB: western blot; IHC: Immunohistochemistry
