# Supplementary material for: Hypomethylation-mediated upregulation of PHOX1 promotes gastric cancer progression via transactivation of NGFR
Source: Cell Death Discov. 2025 Nov 28;11:548. doi: 10.1038/s41420-025-02811-3 (PMC12663246; doi:10.1038/s41420-025-02811-3)

Original, uncropped Western blot images

Figure 2I

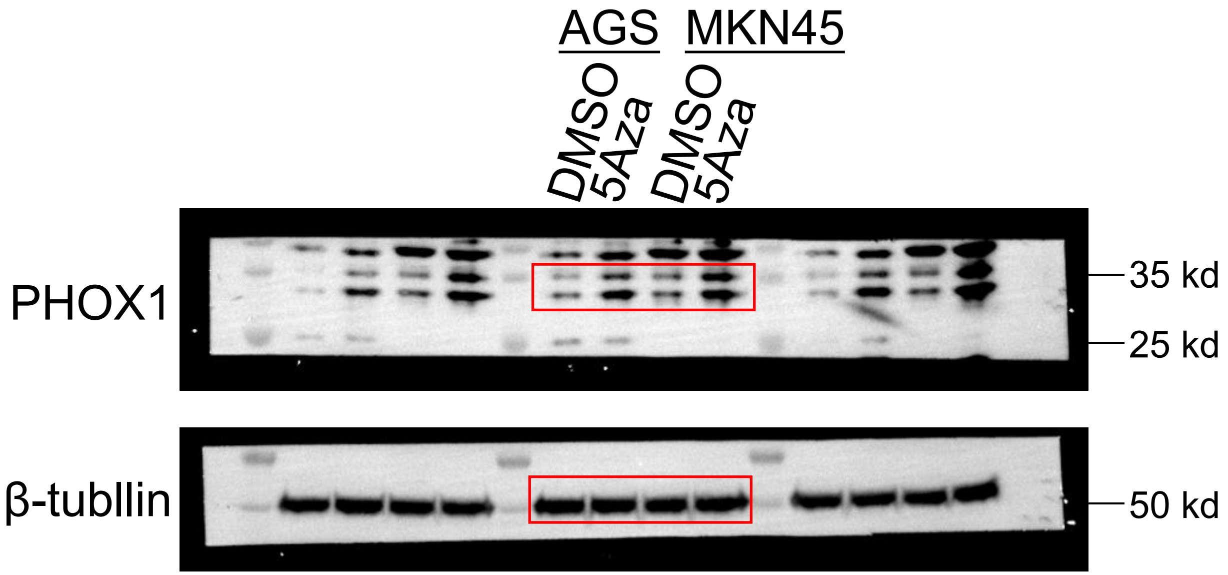

Supplementary Figure S3

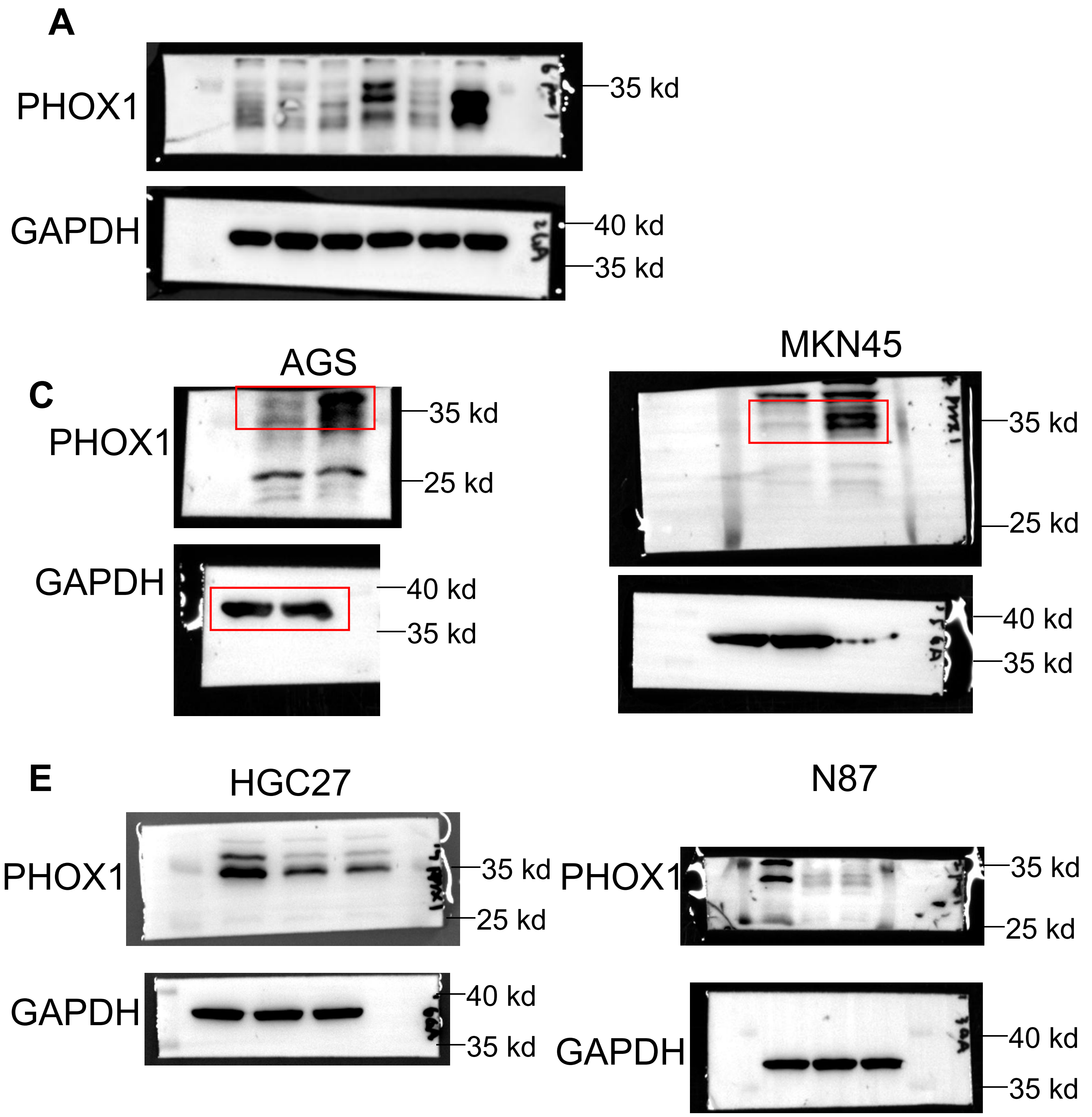

Figure 5F

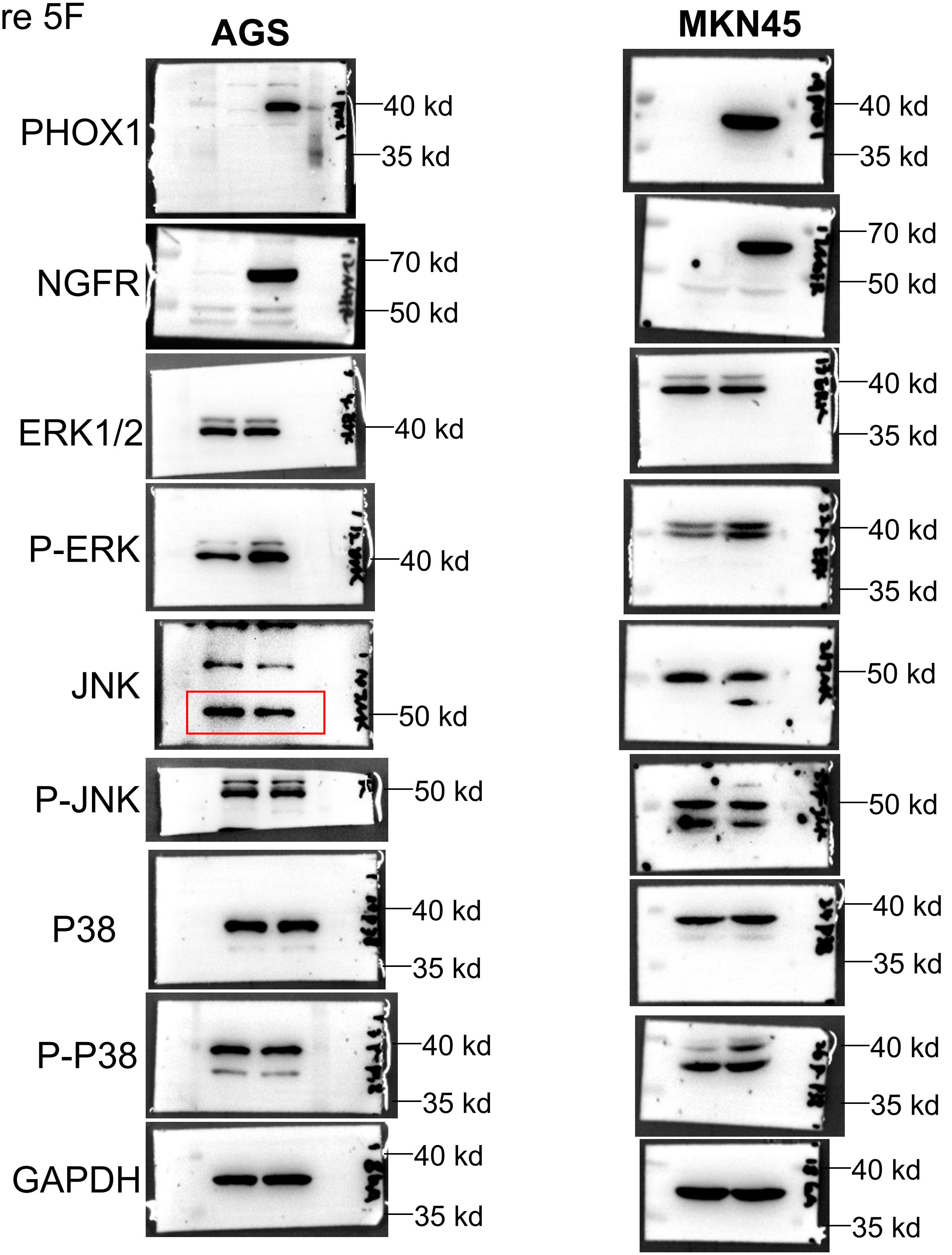
AGS
MKN45
PHOX1
NGFR
ERK1/2
P-ERK
JNK
P-JNK
P38
P-P38
GAPDH
40 kd
35 kd
70 kd
50 kd

Figure 6B

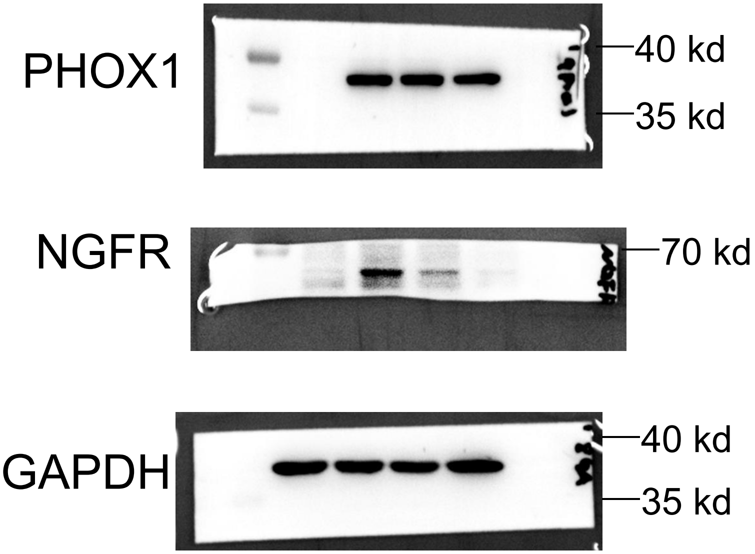

Figure 6D

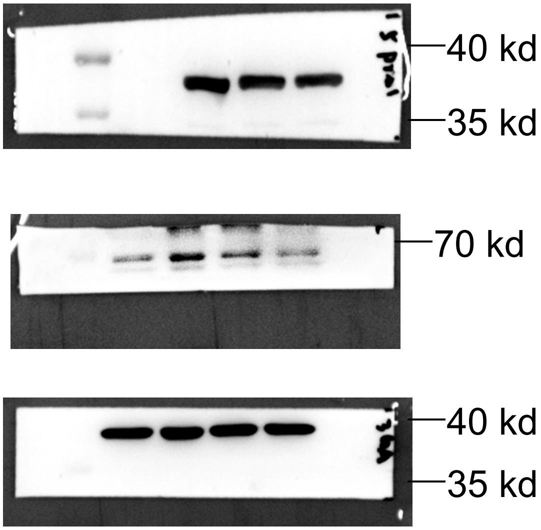

Figure 6L

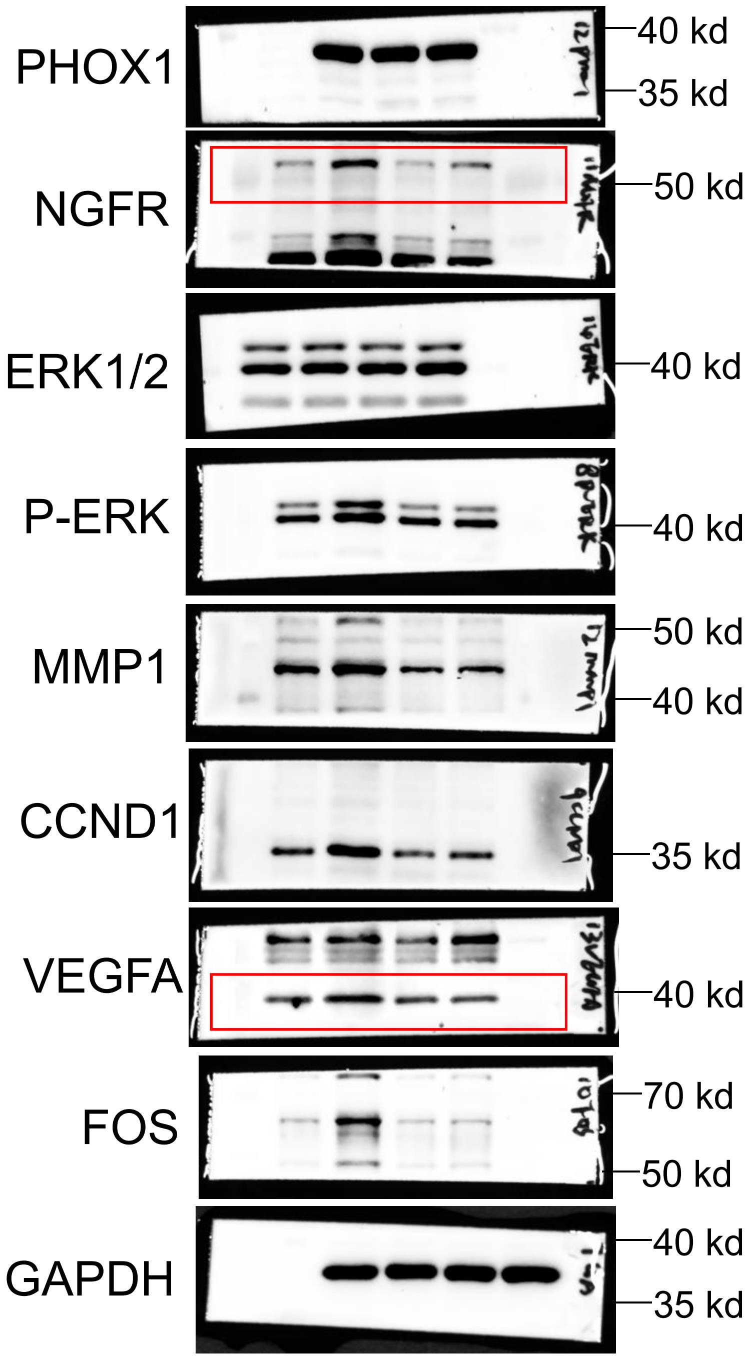

Figure 6M

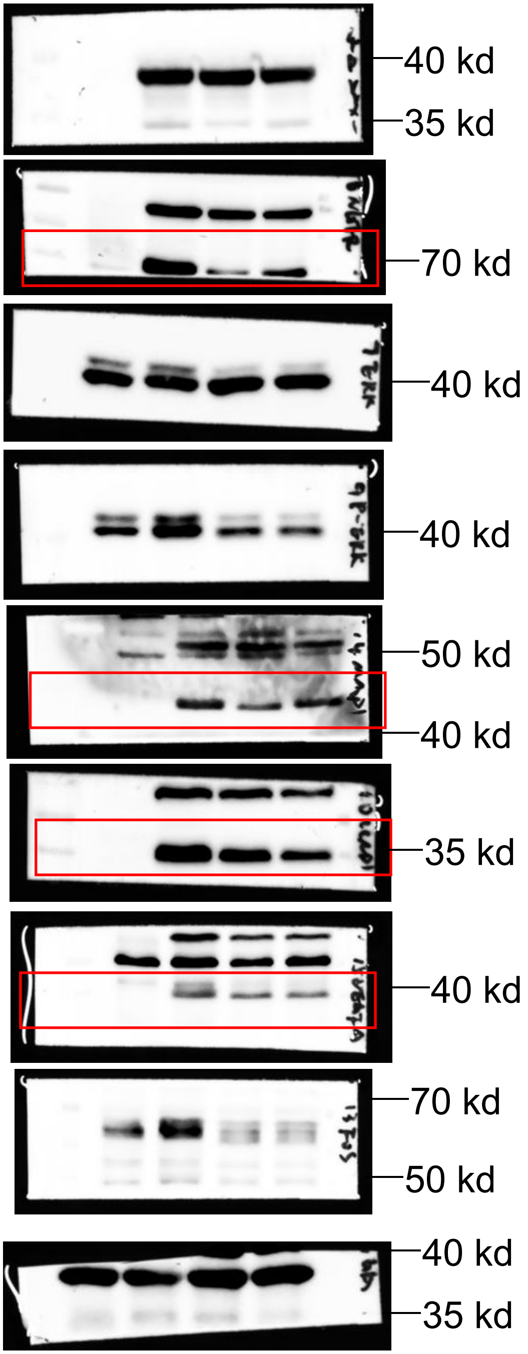

Figure 6N

Figure 6O

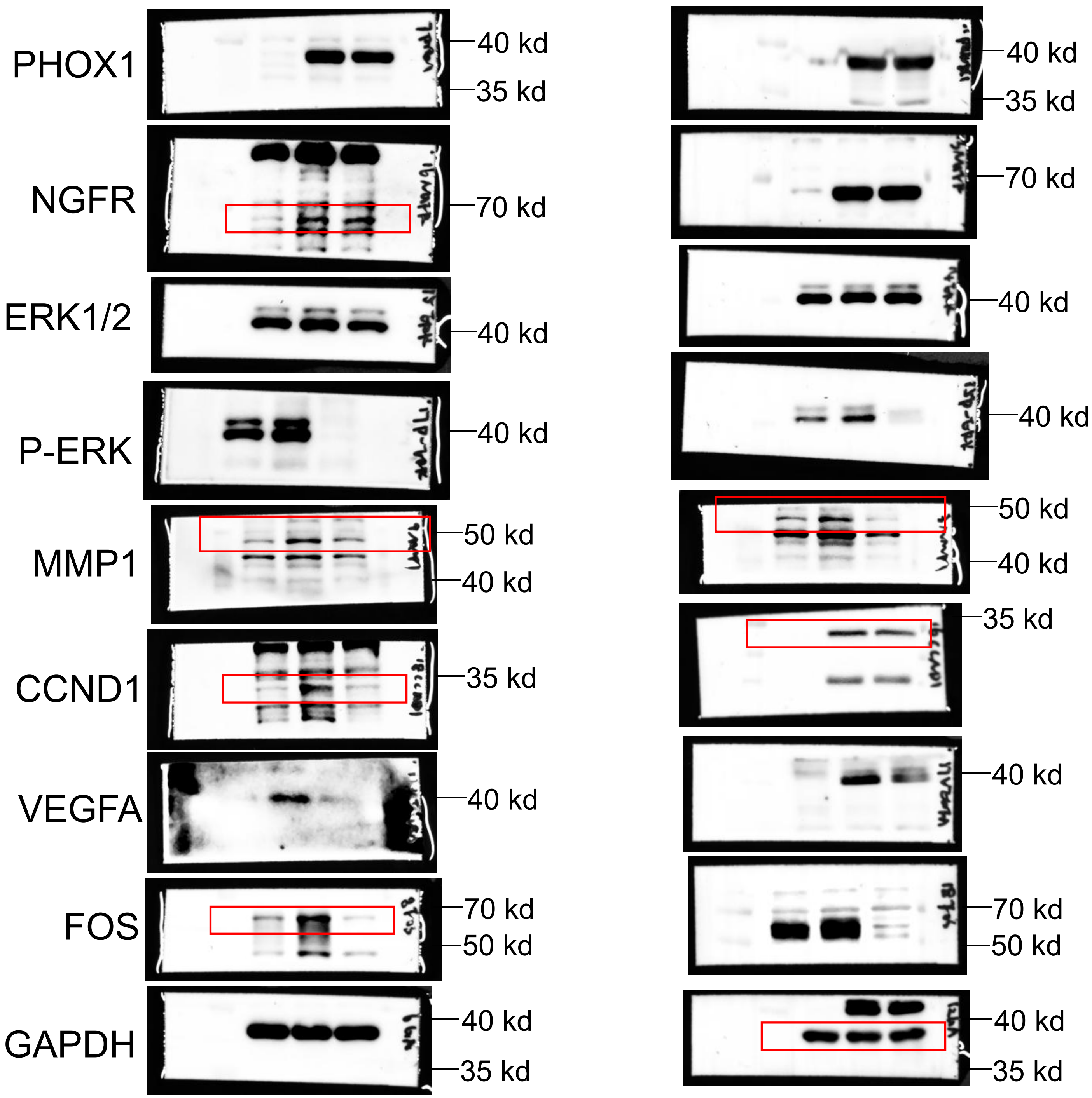

Supplement: Supplementary file 4 — Original WB [file 41420_2025_2811_MOESM4_ESM.pdf]
